# Supplementary material for: Predicting proximal tubule failed repair drivers through regularized regression analysis of single cell multiomic sequencing
Source: Nat Commun. 2024 Feb 12;15:1291. doi: 10.1038/s41467-024-45706-0 (PMC10861555; doi:10.1038/s41467-024-45706-0)
Supplement: Supplementary file 1 — Supplementary Information [file 41467_2024_45706_MOESM1_ESM.pdf]

Supplementary Information

Predicting proximal tubule failed repair drivers through regularized regression analysis of single cell multiomic sequencing

Nicolas Ledru, Parker C. Wilson, Yoshiharu Muto, Yasuhiro Yoshimura, Haojia Wu, Dian Li, Amish Asthana, Stefan G. Tullius, Sushrut S. Waikar, Giuseppe Orlando, and Benjamin D. Humphreys

Table of Contents

Simultaneous single-nucleus transcriptional and chromatin accessibility profiling of the adult human kidney resolves high-quality cell type-specific profiles

Supplementary Figure 1 4

Doublet calling algorithms identify non-overlapping putative doublets

Supplementary Figure 2 5

Partial nephrectomy kidney samples are similar to live donor samples and both contain FR-PT cells

Supplementary Figure 3 6

Identifying *cis*-regulatory elements driving healthy-failed repair PT transition

Supplementary Figure 4 7

Supplementary Figure 5 8

Supplementary Figure 6 9

Supplementary Figure 7 10

Cell type-specific regulatory elements can be predicted with RENIN

Supplementary Figure 8 11

Supplementary Figure 9 12

CRE analysis of healthy-failed repair axis reveals coordinated regulatory element remodeling

Supplementary Figure 10 13

Identification of healthy- and FR-promoting TFs with RENIN

Supplementary Figure 11 14

Supplementary Figure 12 16

Supplementary Figure 13 18

Supplementary Figure 14 19

Supplementary Figure 15 20

Supplementary Figure 16 22

Supplementary Figure 17 23

***NFAT5* knockdown partially reverts failed repair phenotype**

Supplementary Figure 18 24

Supplementary Figure 19 25

Supplementary Figure 20 26

Supplementary Figure 21 27
Supplementary Figure 22 28
Supplementary Figure 23 30
Supplementary Figure 24 31
Supplementary Figure 25 32
Supplementary Figure 26 33

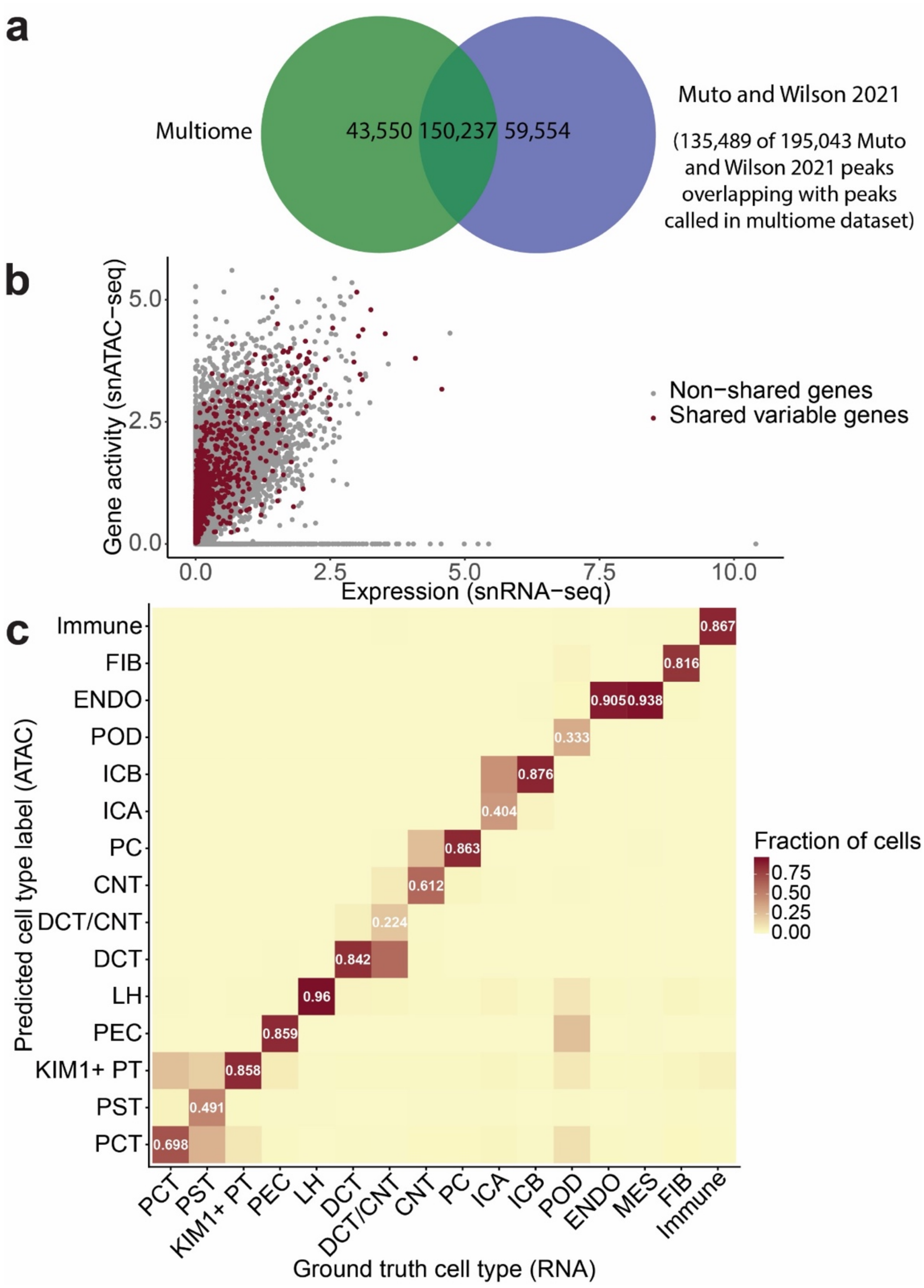

Supplementary Figure 1. Analysis of ATAC modality in single nucleus multiomic dataset. **a.** Overlap between peaks called on this dataset and prior snATAC-seq dataset generated in our lab. **b.** Gene activity scores versus expression for all genes in dataset. Genes that were selected as top variable features in both the Gene Activity and RNA assays are colored red. **c.** Confusion matrix showing agreement between cell type annotations as determined by RNA marker expression and cell type predictions using Seurat's LabelTransfer method.

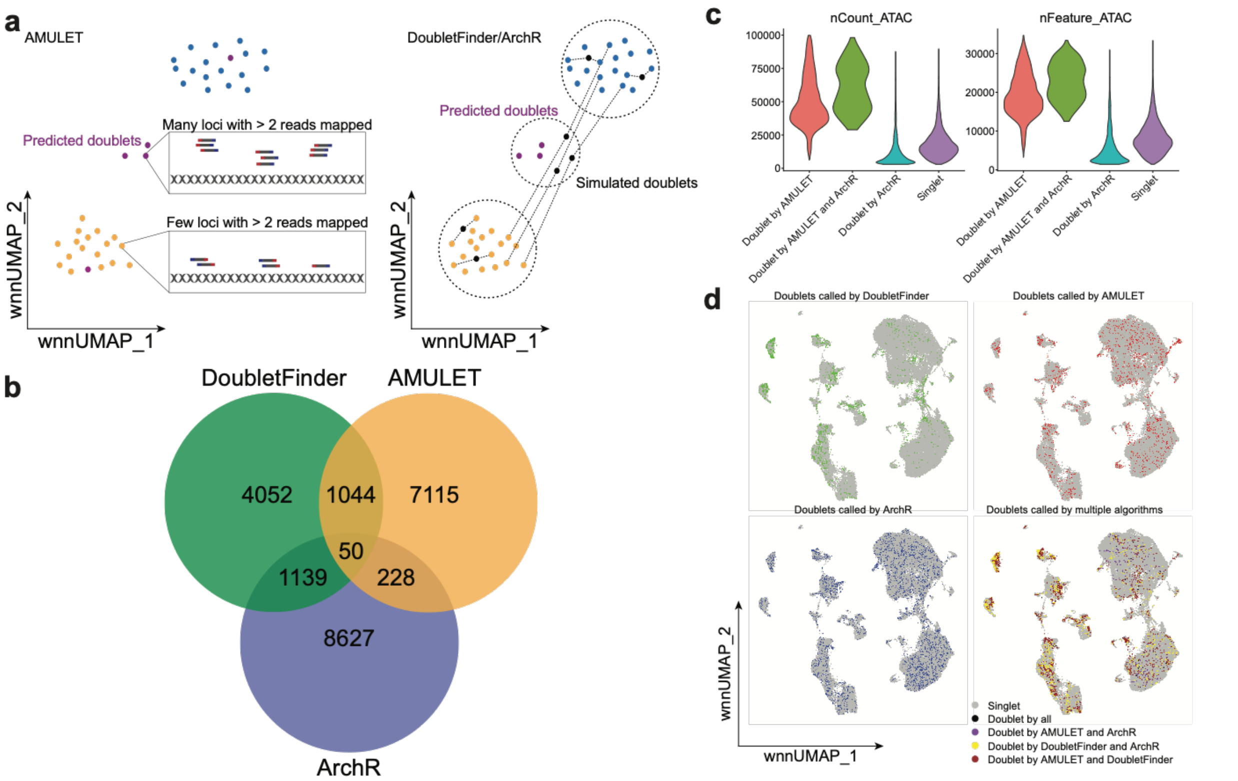

Supplementary Figure 2. Comparison of doublet prediction algorithms. a. Left, cartoon of doublet finding approach used by AMULET. Nuclei have two copies of a given genomic locus, so barcodes with many loci with greater than 2 reads mapped are predicted to be doublets. Right, cartoon of doublet finding approach used by DoubletFinder and ArchR. Doublets are generated by averaging the expression or chromatin accessibility profiles of two barcodes in the dataset. Barcodes are predicted to be doublets by the proportion of neighboring barcodes that are simulated doublets versus original barcodes. **b.** Venn diagram of predicted doublets by each algorithm. **c.** Violin plots for number of unique and total peaks per barcode, labeled by doublet prediction by ArchR and AMULET. **d.** Counterclockwise from top right, WNN UMAP plots of dataset with doublet predictions by AMULET, DoubletFinder, ArchR, and by all three algorithms.

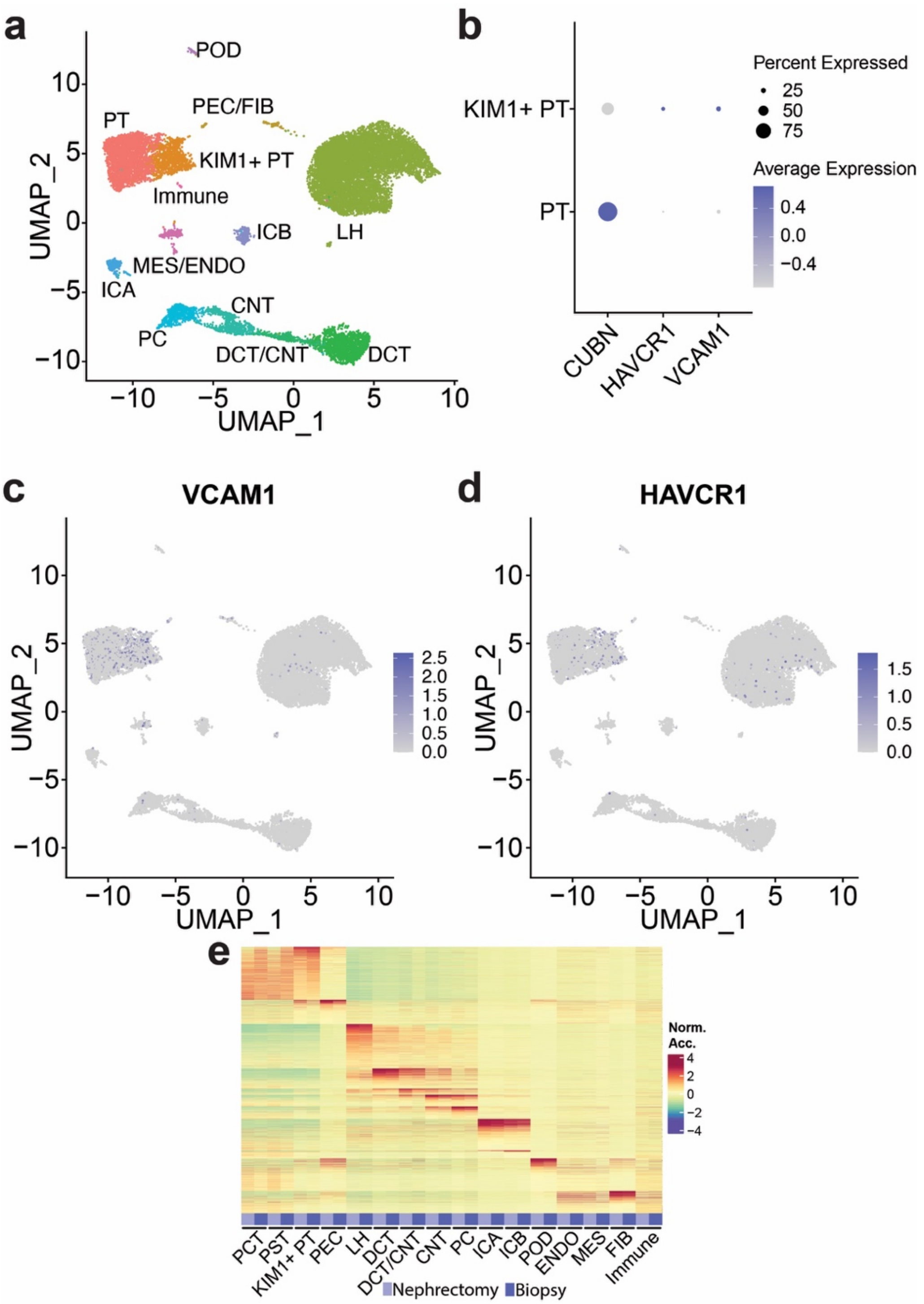

Supplementary Figure 3. KIM1+/VCAM1+ PT population present in living donor biopsy-derived samples. a. UMAP plot of reclustered living donor biopsy-derived-only dataset (n = 5 from 3 individual donors). **b.** Expression dot plot of healthy PT (*CUBN*) and failed repair PT (*VCAM1* and *HAVCR1*) marker genes. **c.** *VCAM1* expression in living donor biopsy-only dataset. **d.** *HAVCR1* expression in living donor biopsy-only dataset. **e.** Heatmap of cell type marker snATAC-seq peak accessibility for each cell type by sample type—nephrectomy or living donor biopsy.

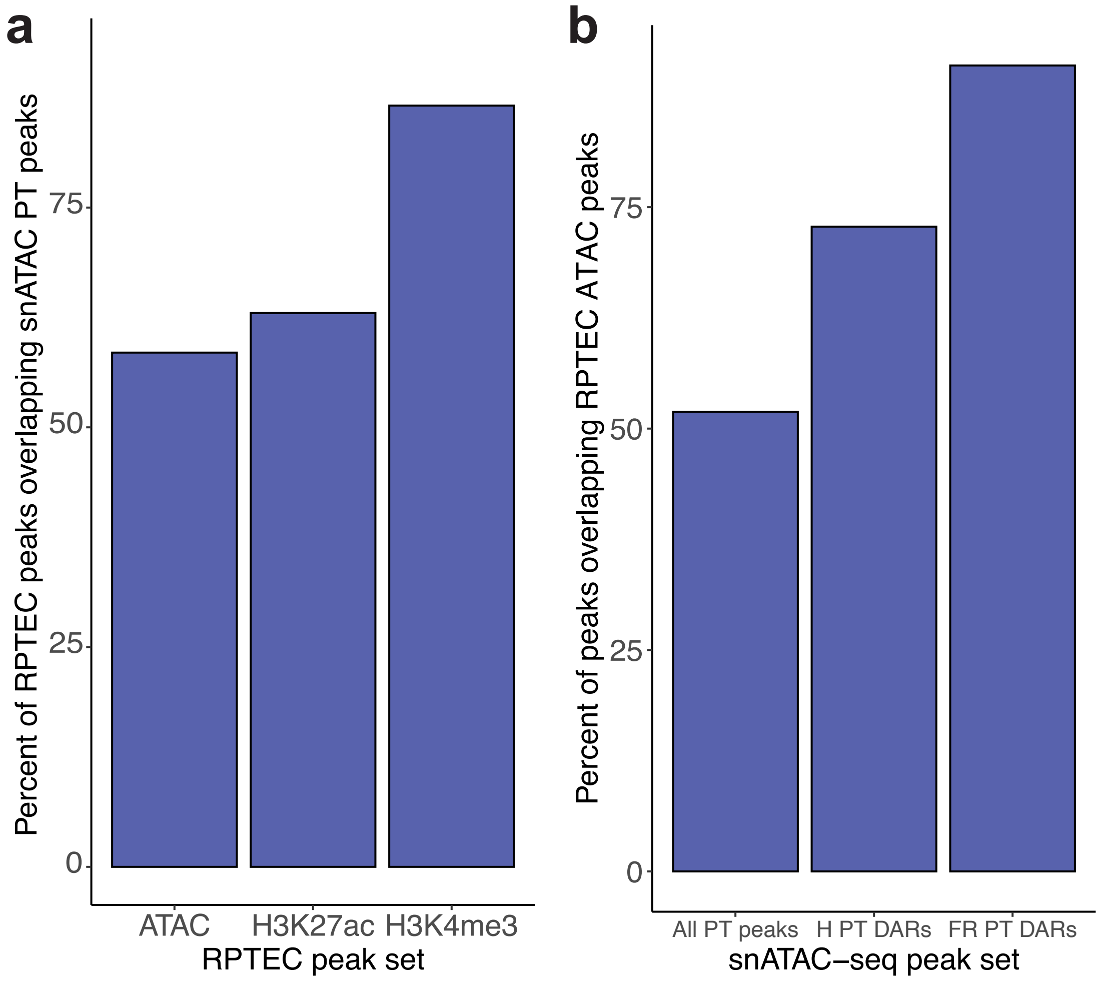

Supplementary Figure 4. Comparison of snATAC-seq and RPTEC chromatin accessibility. a. RPTEC accessible peaks overlap with snATAC PT peaks. 82,414 of 140,850 (58.5%) RPTEC ATAC peaks, 26,199 of 41,599 H3K27ac peaks (63.0%), and 18,547 of 21,407 H3K4me3 peaks (86.6%) overlap accessible chromatin in whole kidney PT accessible peaks. **b.** snATAC-seq PT ATAC-seq overlap with RPTEC ATAC-seq. 75388 of 145340 PT peaks (51.9%), 13467 of 18492 healthy PT DARs (72.8%), and 9179 of 10082 KIM1+ PT peaks (91.0%) overlap RPTEC ATAC-seq peaks.

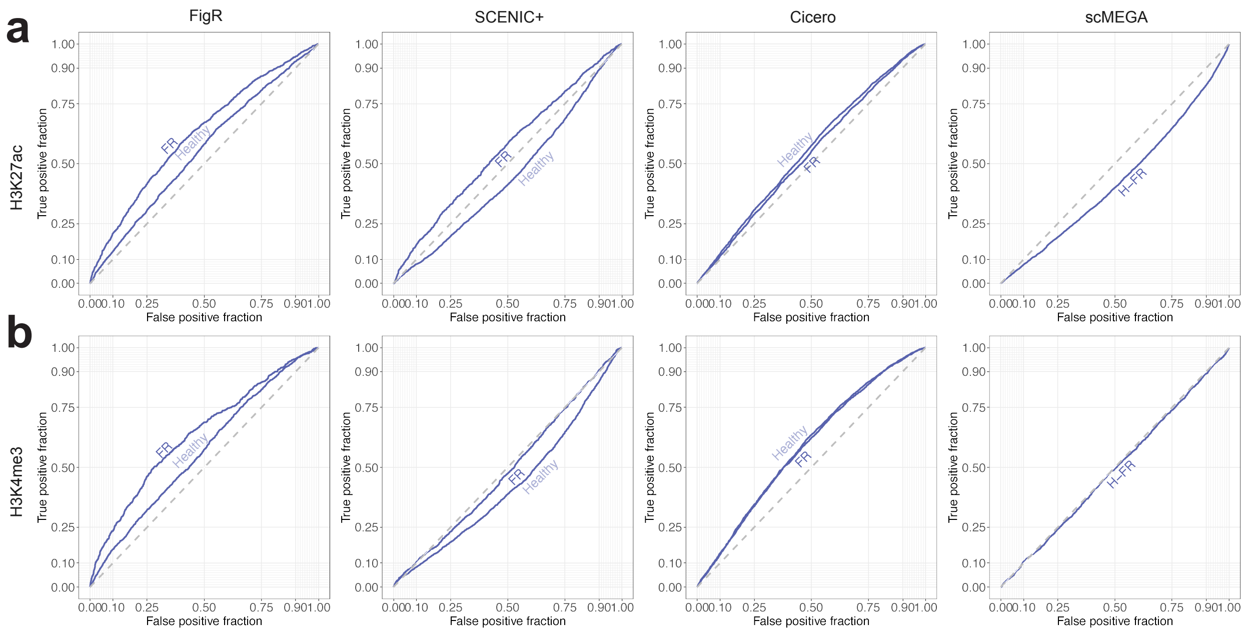

Supplementary Figure 5. RENIN performance measured by healthy and failed repair proximal tubule CREs recall of RPTEC histone modification peaks is competitive with other methods. a. From left to right, ROC curve against RPTEC H3K27ac peaks identified with CUT&RUN, calculated for FigR (FR CRE AUC: 0.622, Healthy CRE AUC: 0.552), SCENIC+ (FR: 0.559, Healthy: 0.450), Cicero (FR: 0.536, Healthy: 0.552), and scMEGA-predicted healthy-FR PT (H-FR AUC: 0.570) CREs. ROC curve for the pseudotime trajectory-based scMEGA is for predicted H-FR trajectory CREs. Dotted line indicates random performance (AUC = 0.5). Source data are provided in the Source Data file. **b.** From left to right, ROC curve calculated against RPTEC H3K4me3 peaks for FigR (FR: 0.637, Healthy: 0.564), SCENIC+ (FR: 0.490, Healthy: 0.428), Cicero (FR: 0.584, H: 0.587), and scMEGA-predicted healthy-FR PT (H-FR AUC: 0.513) CREs. Source data are provided in the Source Data file.

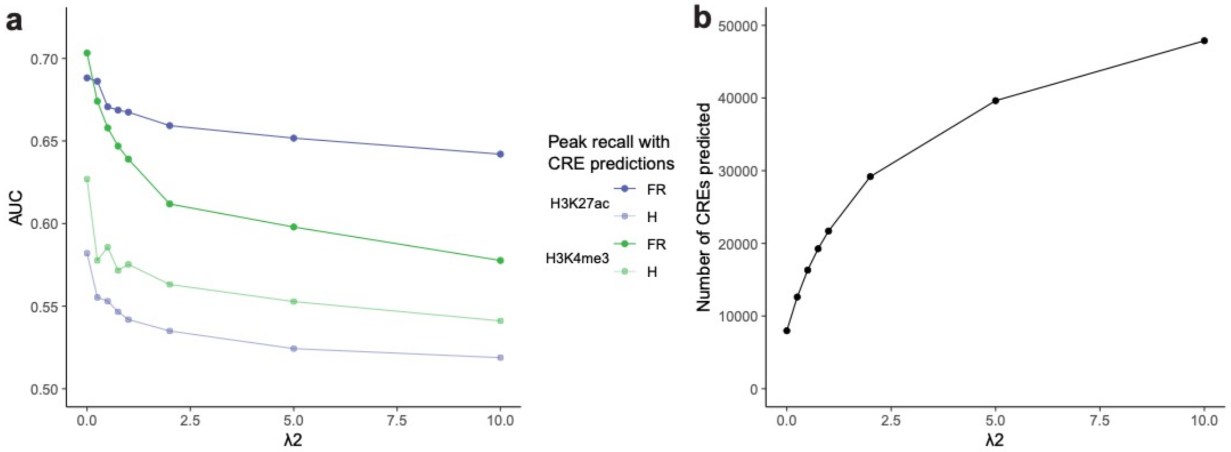

Supplementary Figure 6. The effects of increasing λ_2 on CRE predictions. a. Calculated AUCs for H3K27ac (purple) and H3K4me3 (green) peaks in RPTECs with FR (failed repair PT) and H (healthy PT) CRE predictions with increasing λ_2. **b.** Number of unique CREs predicted with increasing λ_2. Random number generator seeds and pseudocell matrices used were kept the same across trials.

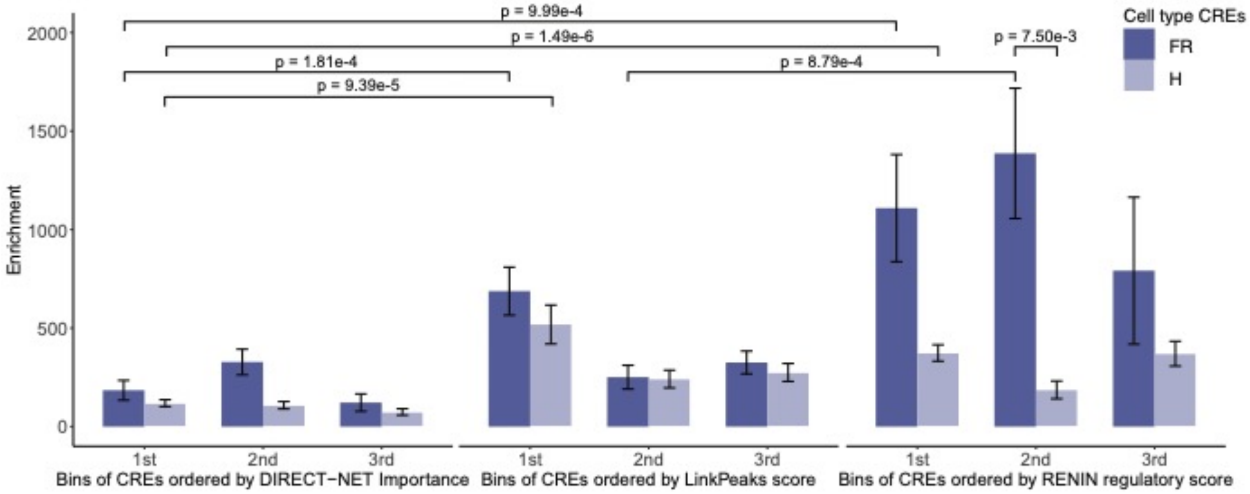

Supplementary Figure 7. RENIN-predicted CREs are enriched for eGFR heritability. Comparison of RENIN, LinkPeaks, and DIRECT-NET by enrichment of partitioned heritability of eGFR in model-predicted healthy (PCT + PST) and FR (failed repair—KIM1+ PT) CREs. P values shown for two-tailed t-test of difference between enrichment means with degrees of freedom = 199. N = 7 biologically independent samples containing 50,768 cells were examined in a joint analysis. Error bars represent standard errors around estimates of enrichment by LDSC with a block jackknife over n=200 equally sized blocks of adjacent SNPs. Source data are provided in the Source Data file.

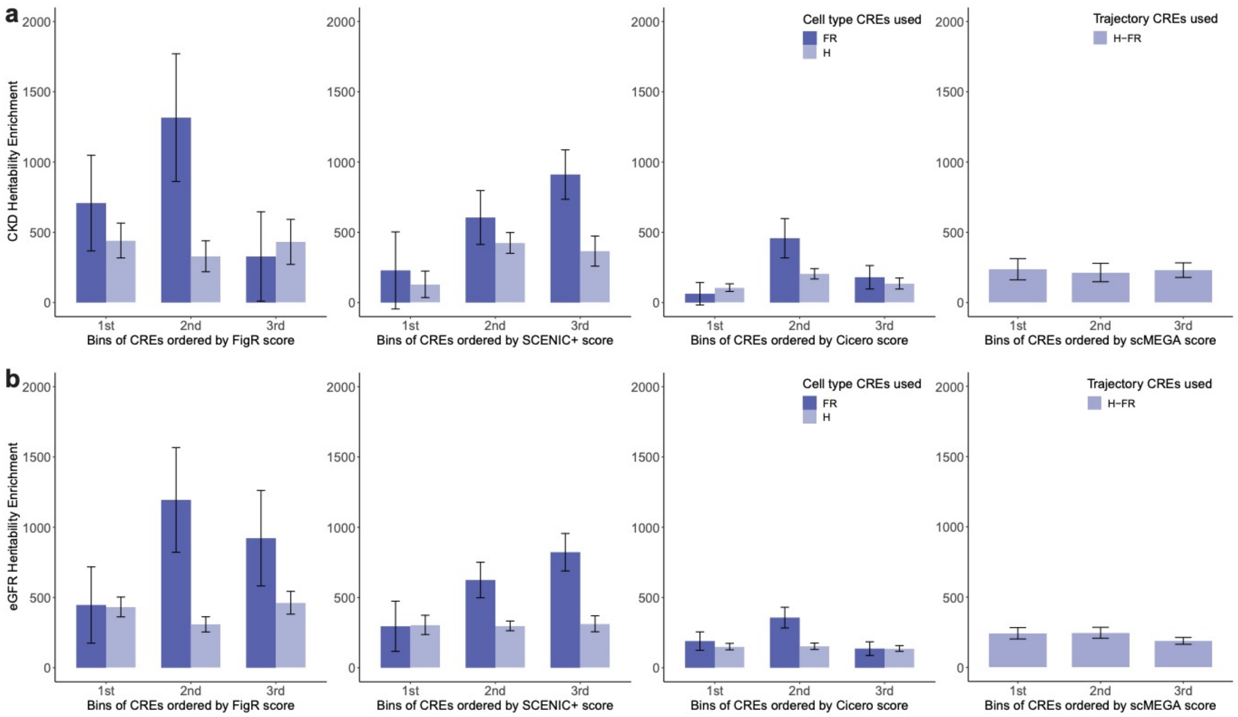

Supplementary Figure 8. From left to right, enrichment of partitioned **(a)** CKD heritability and **(b)** eGFR heritability into predicted CREs by FigR, SCENIC+, Cicero, and scMEGA. To bin model-predicted CREs into top, middle, and bottom third bins, scores calculated by each method were summed for each CRE across all of its target genes. FigR CREs were sorted by rObs, SCENIC+ CREs were sorted by R2G_importance_x_abs_rho, Cicero CREs were sorted by coaccessibility score, and scMEGA CREs were sorted by TStat. N = 7 biologically independent samples containing 50,768 cells were examined in a joint analysis. Error bars represent standard errors around estimates of enrichment by LDSC with a block jackknife over n=200 equally sized blocks of adjacent SNPs. Source data are provided in the Source Data file.

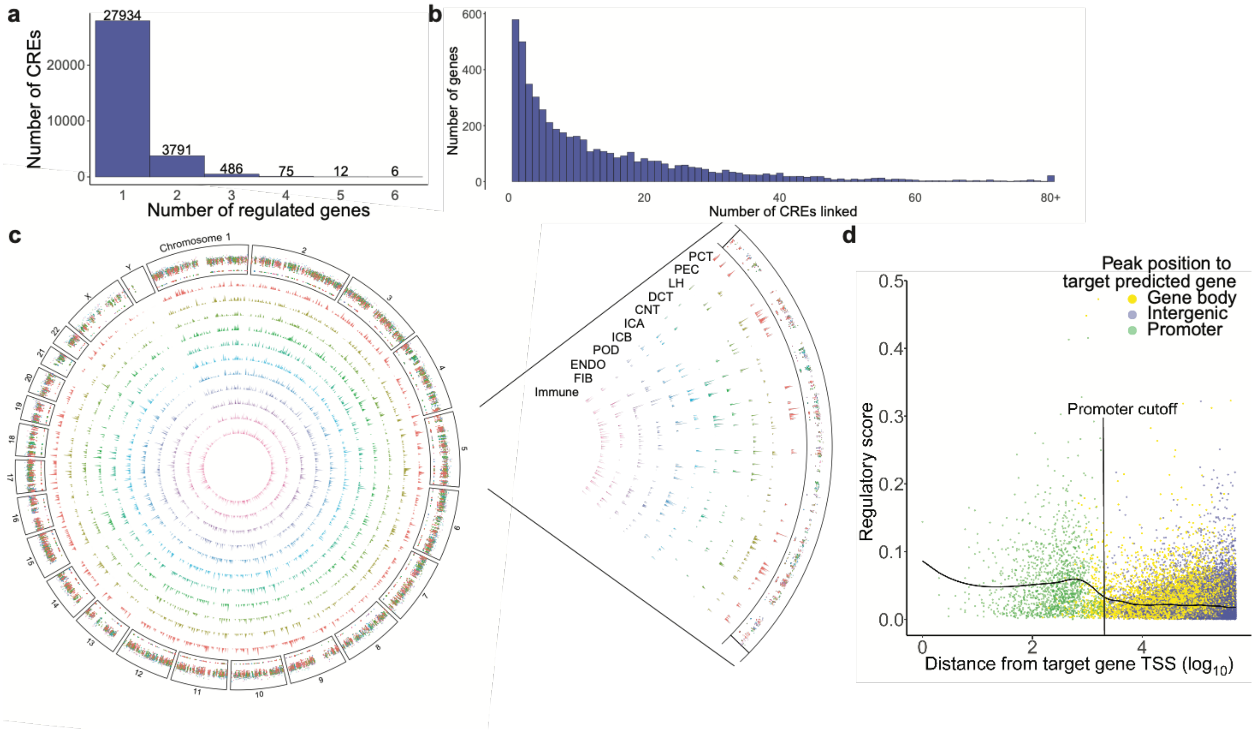

Supplementary Figure 9. a. Histogram showing RENIN-predicted CREs binned by number of predicted linked genes. **b.** Histogram showing modeled cell type marker genes binned by number of RENIN-predicted linked CREs. **c.** Plot of CREs regulating cell type marker genes across genome by cell type. Chromosome 5 is expanded for visualization. **d.** Predicted CRE regulatory score, calculated by summing absolute value of CRE coefficients, by distance from target gene transcription start site (TSS). Peaks falling within target gene promoter are colored green, peaks within target gene body are yellow, and intergenic peaks are purple. Black line is smooth spline curve calculated on data points to illustrate trend over distance.

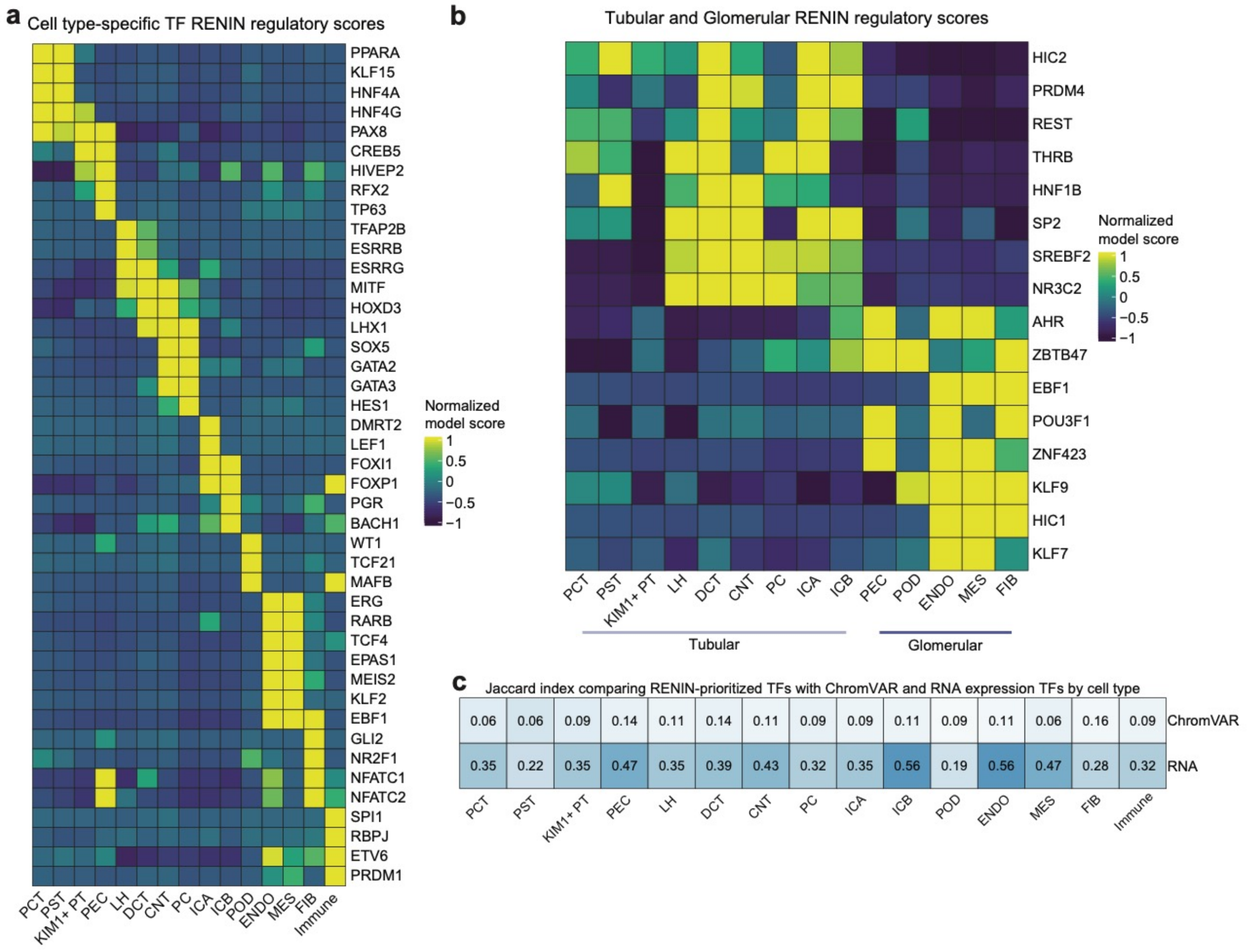

Supplementary Figure 10. **Cell type TF predictions with RENIN. a.** Heatmap of regulatory scores of select TFs by cell type. Regulatory scores calculated by summing regulatory coefficients of a TF on a cell type's marker gene set and multiplying by mean expression of that TF in the given cell type. TF ranks and scores were similar over n = 3 independent trials. **b.** Heatmap of regulatory scores for tubular and glomerular cell type TF predictions. Regulatory scores calculated by summing normalized cell type RENIN scores for tubular or glomerular cell type marker gene set and multiplying by mean expression of that TF in the given cell type. Selected TFs for plotting identified by high summed RENIN scores across tubular (PCT, PST, LH, DCT, CNT, PC, ICA, ICB) or glomerular (POD, PEC, ENDO, MES, FIB) cell types. **c.** Jaccard index between the top 25 TFs for each cell type predicted by RENIN versus ChromVAR enrichment or ranked expression of TFs.

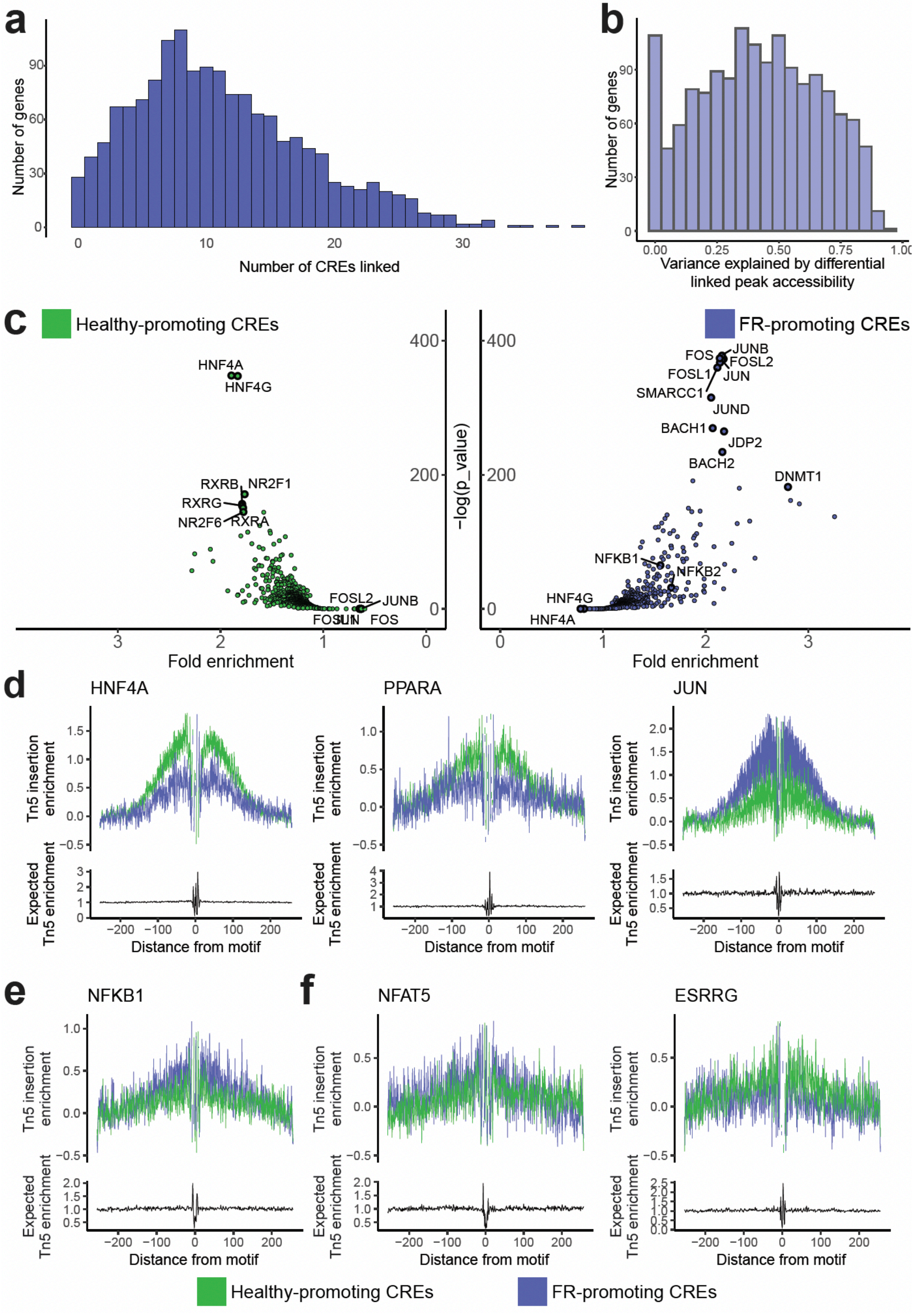
a
Number of genes
Number of CREs linked
b
Number of genes
Variance explained by differential linked peak accessibility
c
Healthy-promoting CREs
FR-promoting CREs
HNF4A
HNF4G
RXRB
NR2F1
RXRG
NR2F6
RXRA
FOSL2
JUNB
FOS
JUN
FOSL1
SMARCC1
JUND
BACH1
JDP2
BACH2
DNMT1
NFKB1
NFKB2
−log(p_value)
Fold enrichment
d
HNF4A
PPARA
JUN
Tn5 insertion enrichment
Expected Tn5 enrichment
Distance from motif
e
NFKB1
f
NFAT5
ESRRG
Healthy-promoting CREs
FR-promoting CREs

Supplementary Figure 11. **Motif analysis of RENIN-predicted CREs regulating healthy-FR PT differentially expressed genes. a.** Histogram showing 1,488 healthy-FR PT modeled genes binned by the number of RENIN-predicted linked CREs. **b.** Histogram of coefficients of determination of model predictions of DEG expression versus actual expression when training model to use linked CRE accessibility as predictor variables. **c.** Motif analysis of CREs predicted to regulate differentially expressed genes between healthy PT (PCT and PST) and FR PT (KIM1+ PT) clusters. Left, healthy-promoting CREs were identified by predicted positive regulatory score on genes upregulated in healthy PT compared to FR PT or negative regulatory score on genes upregulated in FR PT compared to healthy PT. Right, FR-promoting CREs identified by positive score on genes upregulated in FR PT or negative score on genes upregulated in healthy PT. **d.** Footprinting analysis for HNF4A, left; PPARA, middle; JUN, right. Tn5 insertion enrichment calculated around motifs present in healthy-promoting CREs (green) and motifs present in FR-promoting CREs (purple). **e**. Footprinting analysis for NFKB1. **f.** Footprinting analysis for NFAT5, left, and ESRRG, right. For **d-f**, n = 7 biologically independent samples containing 50,768 cells were examined in a joint analysis. Tn5 insertion enrichment calculated around motifs present in healthy-promoting CREs (green) and motifs present in FR-promoting CREs (purple).

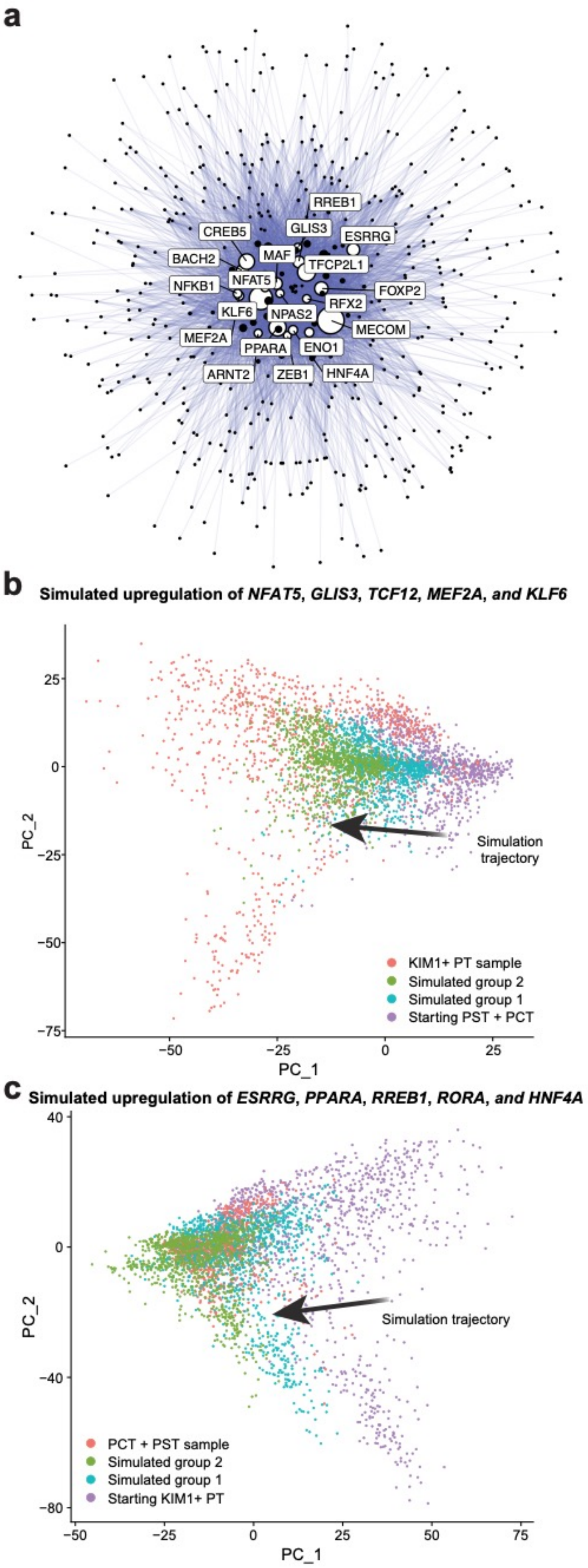
a
RREB1
GLIS3
ESRRG
CREB5
MAF
TFCP2L1
BACH2
NFAT5
FOXP2
NFKB1
RFX2
KLF6
NPAS2
MECOM
MEF2A
PPARA
ENO1
ARNT2
ZEB1
HNF4A
b
Simulated upregulation of NFAT5, GLIS3, TCF12, MEF2A, and KLF6
Simulation trajectory
KIM1+ PT sample
Simulated group 2
Simulated group 1
Starting PST + PCT
PC_2
PC_1
c
Simulated upregulation of ESRRG, PPARA, RREB1, RORA, and HNF4A
Simulation trajectory
PCT + PST sample
Simulated group 2
Simulated group 1
Starting KIM1+ PT
PC_2
PC_1

Supplemental Figure 12. Visualization of gene regulatory networks predicted by RENIN by graph and simulation. a. TF node size represents centrality computed by betweenness, top 20 TFs are labeled. **b.** PCA plot of subsampled PT dataset, with n = 1000 KIM1+ PT cells and n = 1000 starting PST + PCT cells. Simulated group 1 and 2 are results of upregulating *NFAT5*, *GLIS3*, *TCF12*, *MEF2A*, and *KLF6* in the starting PST + PCT subset. **c.** PCA plot of subsampled PT dataset, with n = 1000 starting KIM1+ PT cells and n = 1000 PST + PCT cells. Simulated group 1 and 2 are results of upregulating *ESRRG*, *PPARA*, *RREB1*, *RORA*, and *HNF4A* in the starting KIM1+ subset. TF upregulation is simulated by increasing expression by 5 (group 1) and 10 (group 2) standard deviations in the starting sample, calculated by each TF's expression in the PT dataset. Simulation results were similar over n = 3 independent trials.

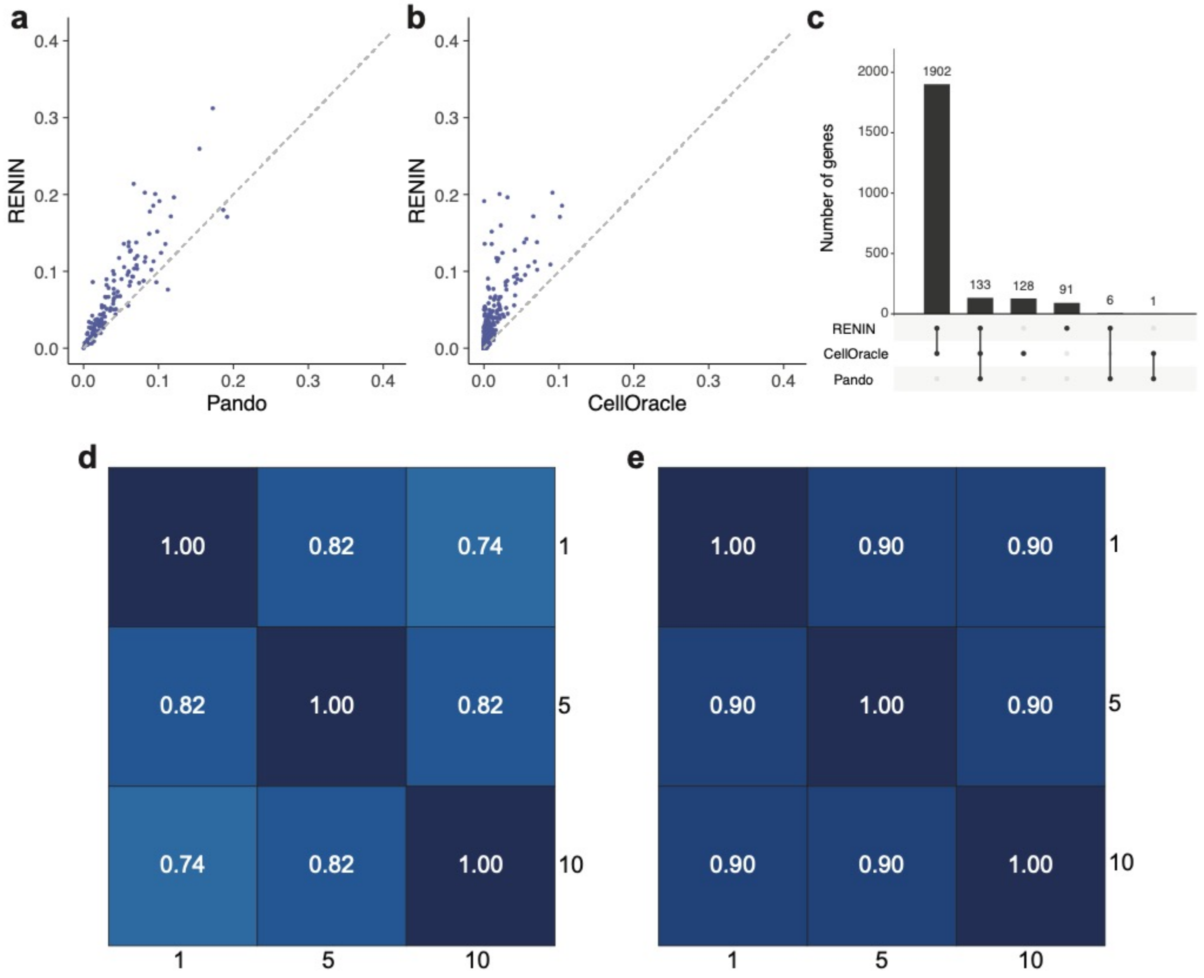

Supplementary Figure 13. Benchmarking RENIN predictions. a. r^2 calculated for RENIN- and Pando-predicted H-FR gene expression by training on Gerhardt 2023 multiomic dataset compared to target gene expression in an independent Kirita 2020 dataset for genes that were successfully modeled by both methods. For shared genes, mean RENIN r^2 was .068 and mean Pando r^2 was .042. Source data are provided in the Source Data file. **b.** r^2 calculated for RENIN- and CellOracle-predicted H-FR gene expression by training on Gerhardt 2023 multiomic dataset compared to target gene expression in an independent Kirita 2020 dataset for genes that were successfully modeled by both methods. For shared genes, mean RENIN r^2 was .012 and mean CellOracle r^2 was .0035. Source data are provided in the Source Data file. **c.** Number of H-FR differentially expressed genes modeled by each method by training on Gerhardt 2023 multiomic dataset. Source data are provided in the Source Data file. **d-e.** To measure similarity of TF predictions using different sizes of pseudocells, matrix of pairwise Jaccard indexes calculated for the top 20 model predicted **(d)** FR and **(e)** H TFs using target bin sizes of 1 (no pseudocell binning), 5, 10 cells per pseudocell.

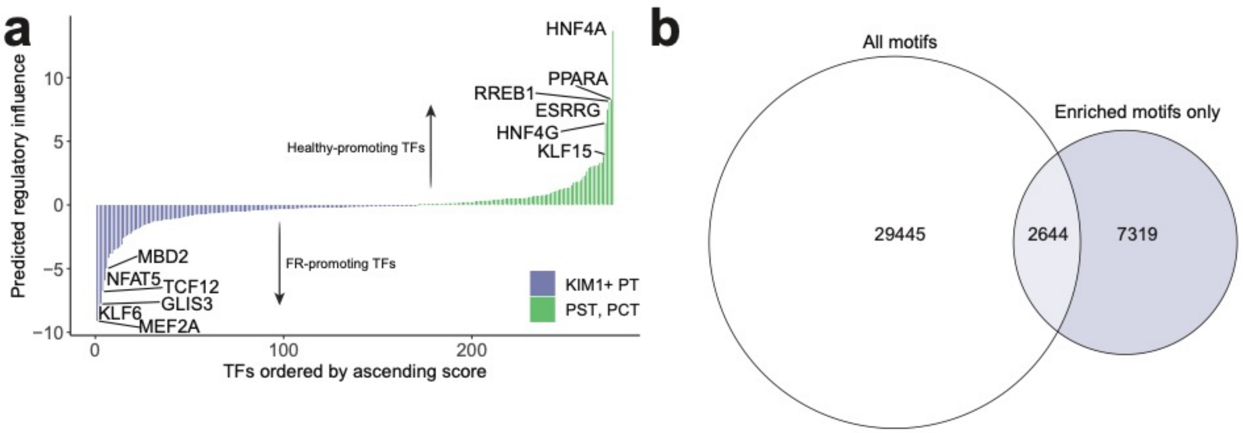

Supplementary Figure 14. Testing the effects of using motif enrichment for modeling. a. TFs sorted by predicted regulatory score. For each modeled H-FR differentially expressed gene, TFs with significantly enriched motifs within its set of linked CREs were identified and used for regression modeling. Similar TF rankings and scores replicated over n = 3 independent trials. **b.** Overlap of unique predicted TF-gene links between the two modeling configurations. For the All motifs configuration, any motif appearing at least once in a gene's linked CREs is considered. For the Enriched motifs only configuration, only TFs with significantly enriched motifs are considered.

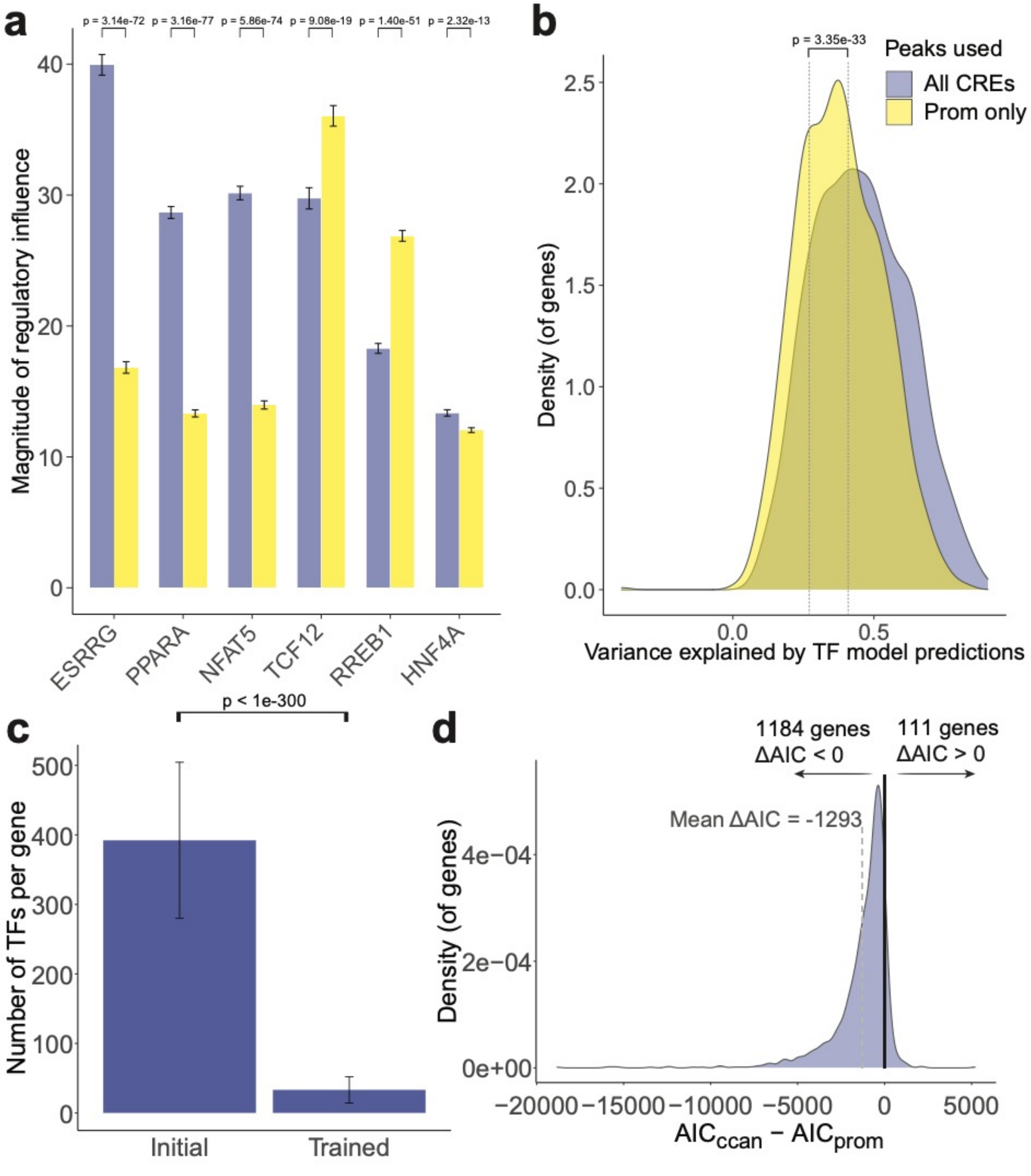
a
p = 3.14e-72
p = 3.16e-77
p = 5.86e-74
p = 9.08e-19
p = 1.40e-51
p = 2.32e-13
Magnitude of regulatory influence
0
10
20
30
40
ESRRG
PPARA
NFAT5
TCF12
RREB1
HNF4A
b
p = 3.35e-33
Peaks used
All CREs
Prom only
Density (of genes)
0.0
0.5
1.0
1.5
2.0
2.5
Variance explained by TF model predictions
c
p < 1e-300
Number of TFs per gene
0
100
200
300
400
500
Initial
Trained
d
1184 genes
ΔAIC < 0
111 genes
ΔAIC > 0
Mean ΔAIC = -1293
Density (of genes)
0e+00
2e−04
4e−04
−20000
−15000
−10000
−5000
0
5000
AICccan − AICprom

Supplementary Figure 15. Comparing the use of promoters and distal CREs for modeling. a. Regulatory scores calculated for selected TFs when model is trained with motifs from all linked CREs (purple) and from motifs only in target gene's promoter region (yellow). P values shown for two-tailed t-test of the difference in mean scores. Data presented as mean estimates of regulatory scores with error bars representing standard errors, calculated by modeling n=100 bootstrapped samples. Source data are provided in the Source Data file. **b.** Density plot of coefficients of determination of model predictions of differentially expressed gene expression versus actual expression when training model with motifs from all linked CREs (purple, mean $r^2 = 0.45$) and motifs only in target gene's promoter region (yellow, mean $r^2 = 0.38$). P value calculated for two-tailed two sample t-test. Source data are provided in the Source Data file. **c.** Number of TFs per modeled gene before and after RENIN training. The initial number consists of all unique TF motifs present in a gene's linked CREs and promoter peaks. The trained number of TFs consists of the number of TFs with RENIN-predicted non-zero regulatory weights for that gene. P value calculated for two-tailed paired t-test. Analysis performed for n=1514 modeled genes. Source data are provided in the Source Data file. **d.** Density plot of ΔAIC for 1323 modeled genes with at least one promoter peak (MACS2-called peak within 2000bp of TSS), calculating ΔAIC as the difference between the AIC of the RENIN-trained model using TFs within all predicted linked CREs (CCAN—*cis*-coaccessibility network) and the AIC of the trained model using TFs within the promoter peak(s) only. Mean (gray dashed line) and median ΔAIC are -1293 and -771, respectively. 1184 genes have a negative ΔAIC, while 111 have a positive ΔAIC (black line).

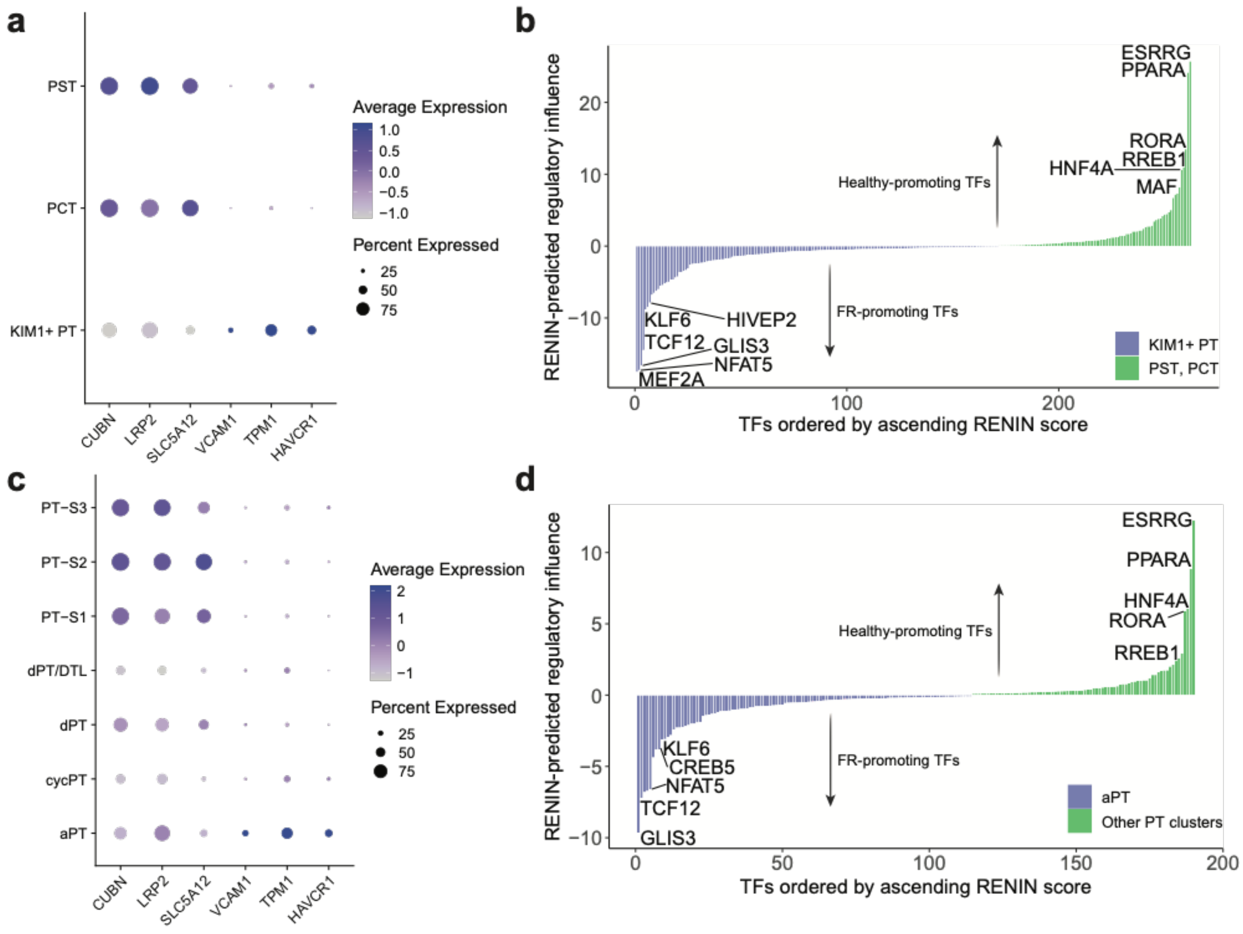

Supplementary Figure 16. **Modeling H-FR transition in other human datasets. a.** Expression of healthy PT (*CUBN*, *LRP2*, *SLC5A12*) and FR PT (*VCAM1*, *TPM1*, *HAVCR1*) markers by cell type annotation in merged n=15 snRNA-seq dataset. **b.** TFs sorted by regulatory score, calculated by applying RENIN to merged n=15 snRNA-seq dataset. FR-healthy comparison was between KIM1+ PT-labeled cells and PST- or PCT-labeled cells. **c.** Expression of healthy PT (*CUBN*, *LRP2*, *SLC5A12*) and FR PT (*VCAM1*, *TPM1*, *HAVCR1*) markers by cell type annotation in merged n=27 snRNA-seq KPMP dataset. **d.** TFs sorted by regulatory score, calculated by applying RENIN to n=27 snRNA-seq KPMP dataset. FR-healthy comparison was between the aPT cluster and all other PT clusters. Similar TF rankings and scores replicated over n = 3 independent trials.

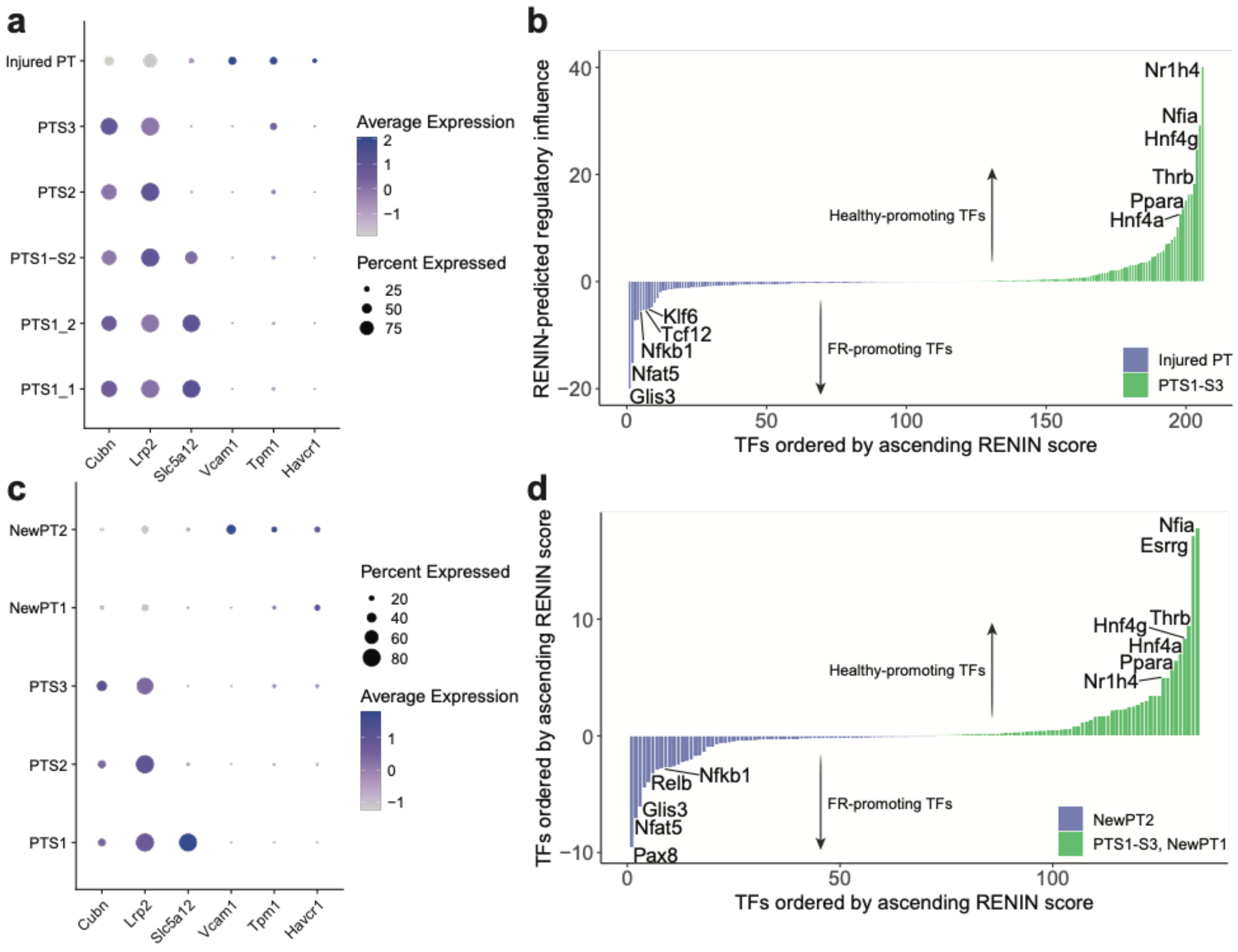

Supplementary Figure 17. **Modeling H-FR transition in other mouse datasets. a.** Expression of healthy PT (*Cubn*, *Lrp2*, *Slc5a12*) and FR PT (*Vcam1*, *Tpm1*, *Havcr1*) markers by cell type annotation in Gerhardt 2023 multiomic dataset. **b.** TFs sorted by regulatory score, calculated by applying RENIN to Gerhardt 2023 multiomic dataset. FR-healthy comparison was between Injured PT-labeled cells and PTS1, PTS2, and PTS3-labeled cells. **c.** Expression of healthy PT (*Cubn*, *Lrp2*, *Slc5a12*) and FR PT (*Vcam1*, *Tpm1*, *Havcr1*) markers by cell type annotation in Kirita 2020 snRNA-seq dataset. **d.** TFs sorted by regulatory score, calculated by applying RENIN to Kirita 2020 snRNA-seq dataset. FR-healthy comparison was between the NewPT2 cluster and all other PT clusters. Similar TF rankings and scores replicated over n = 3 independent trials.

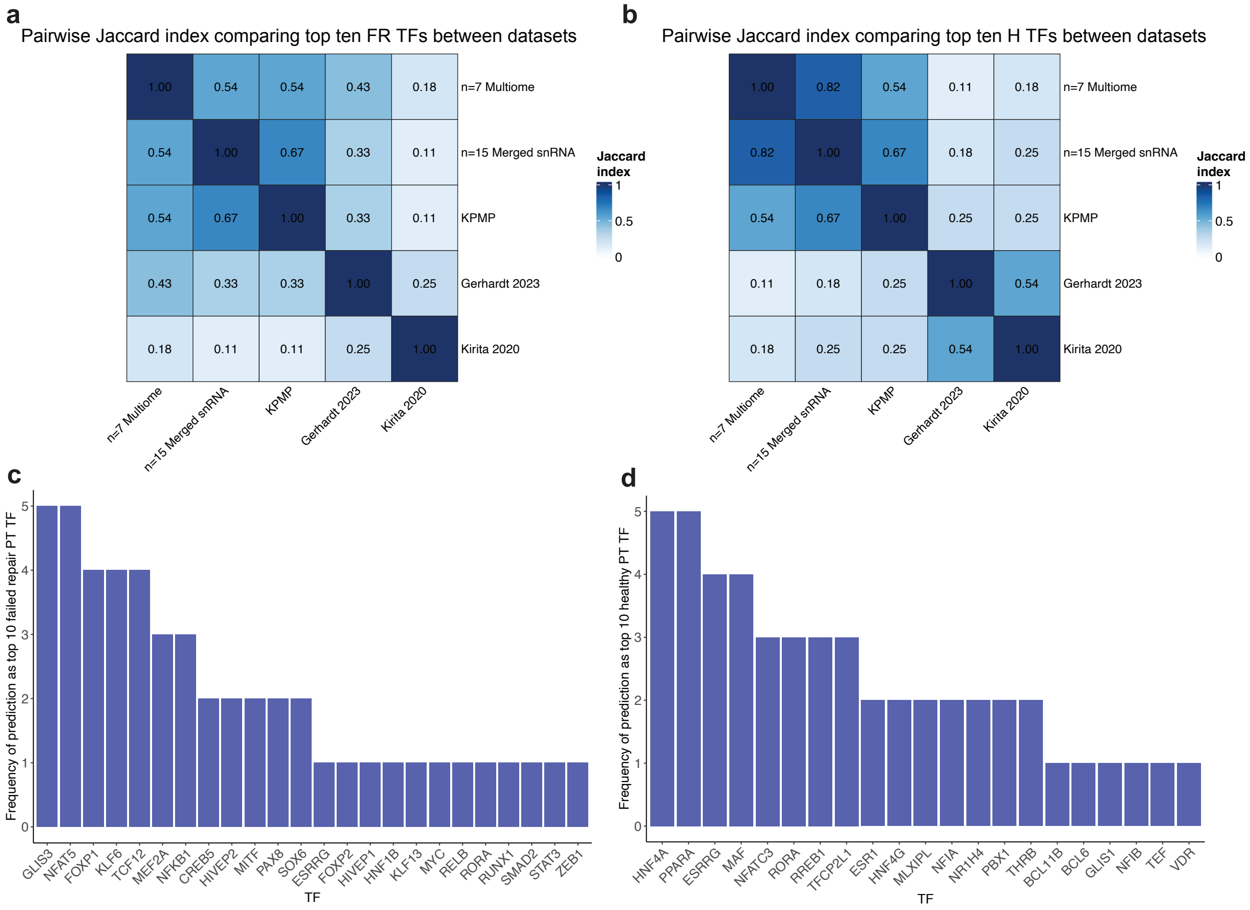

Supplementary Figure 18. **Comparison of predictions between five modeled datasets. a.** Matrix of Jaccard indexes calculated between the top ten predicted FR-associated TFs for each dataset. Index of 1 indicates perfect overlap and index of 0 indicates no overlap. **b.** Matrix of Jaccard indexes calculated between the top ten predicted healthy-associated TFs for each dataset. Index of 1 indicates perfect overlap and index of 0 indicates no overlap. **c.** Frequency of prediction as a top ten FR-associated TF for all TFs in at least one top 10 FR list. TFs that were top FR predictions in all 5 modeled datasets are GLIS3 and NFAT5. **d.** Frequency of prediction as a top ten healthy-associated TF for all TFs in at least one top 10 healthy list. TFs that were top healthy predictions in all 5 modeled datasets are HNF4A and PPARA.

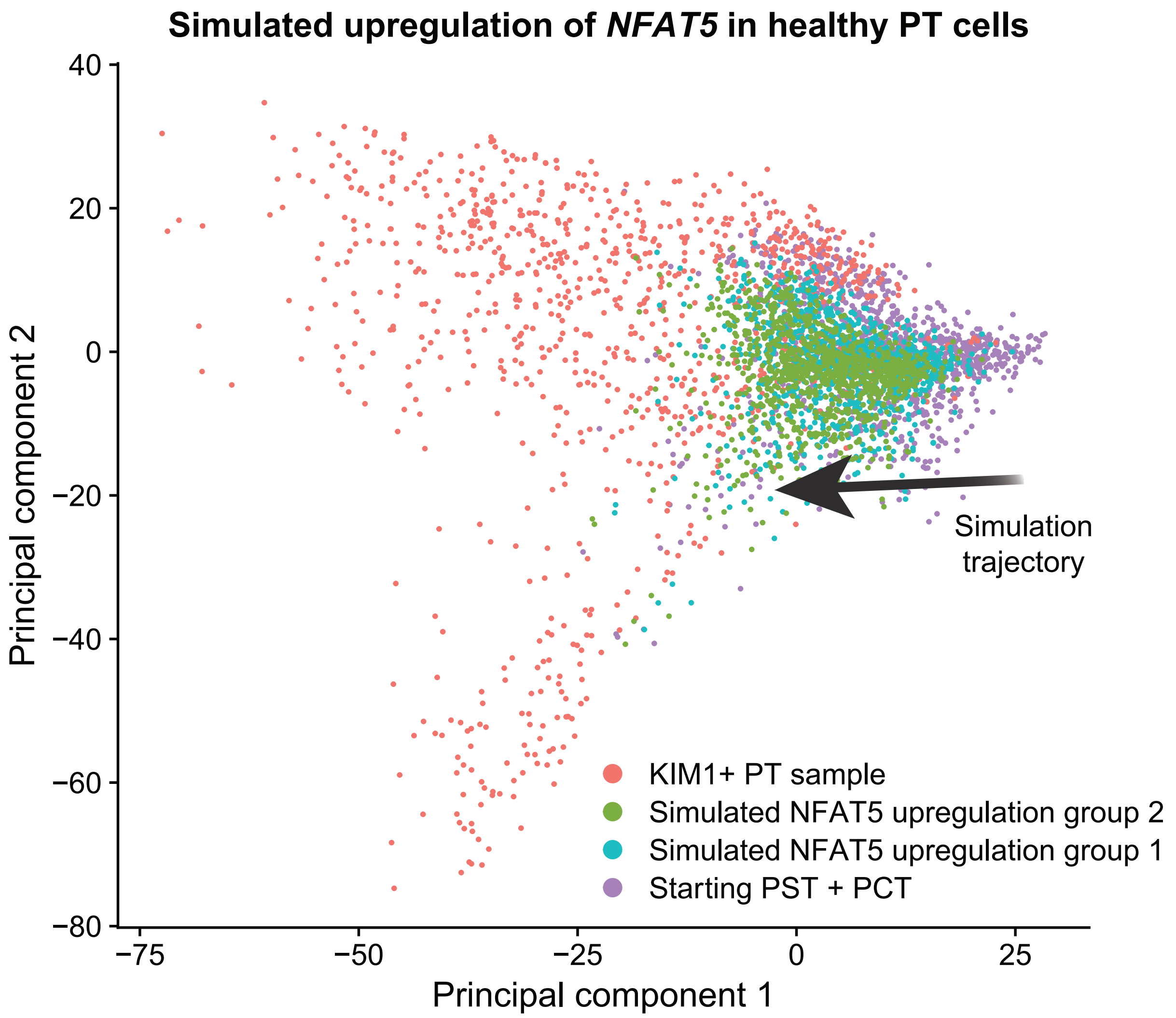

Supplementary Figure 19. Simulated NFAT5 upregulation in PST and PCT cells increases FR expression phenotype. PCA plot of subsampled PT dataset, with n = 1000 starting PST and PCT cells and n = 1000 KIM1+ PT cells. Upregulation of *NFAT5* is simulated in these starting healthy PST and PCT cells (Simulated group 1 and 2). Simulated group 1 and 2 are results of upregulating *NFAT5* by 5 (group 1) and 10 (group 2) standard deviations in the starting PST + PCT sample, calculated by each TF's expression in the PT dataset. Simulation results were similar over n = 3 independent trials.

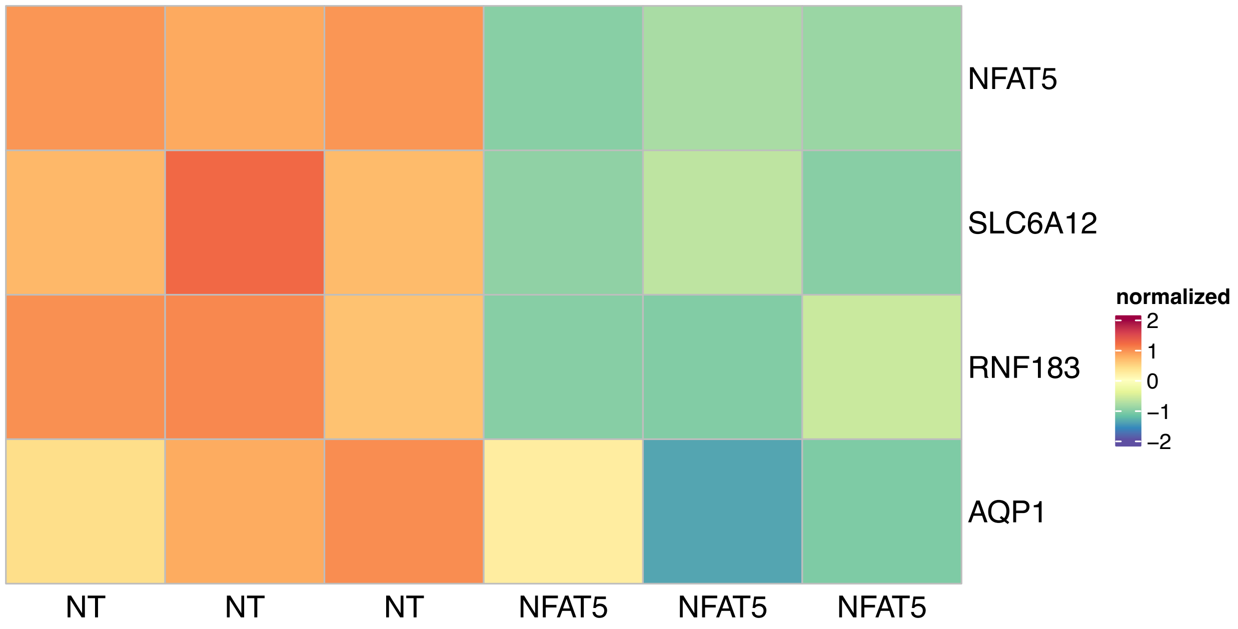

Supplementary Figure 20. Known targets of NFAT5 are significantly downregulated with NFAT5 knockdown in RPTEC culture. SLC6A12 (BGT1), RNF183, and AQP1 are significantly downregulated with *NFAT5* siRNA treatment. DESeq2-calculated adjusted p values: 1.293e-3, 1.241e-14, and 3.525e-02, respectively. Comparison performed between n = 3 independent non-targeting control samples and n = 3 independent *NFAT5*-targeting siRNA samples.

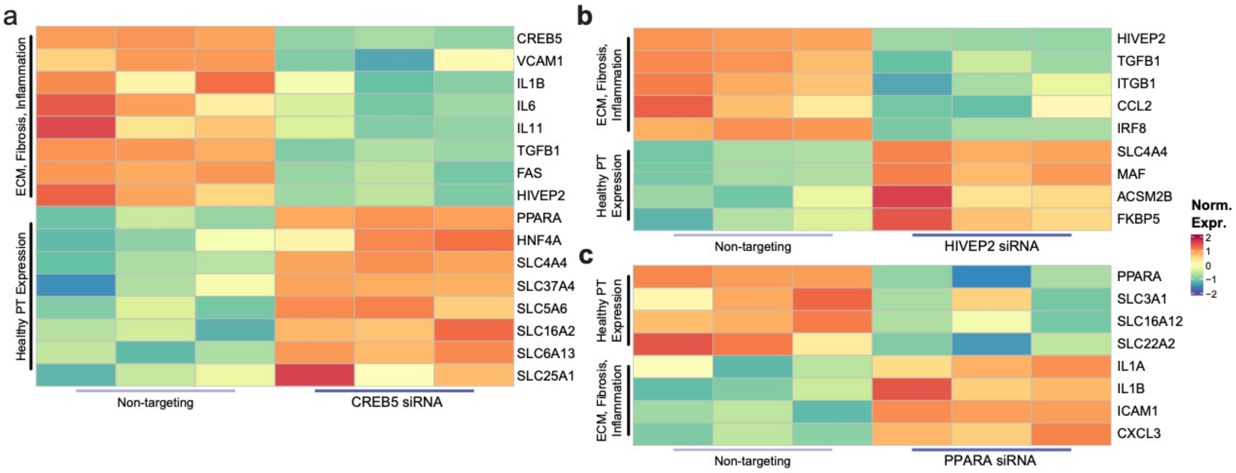

Supplementary Figure 21. siRNA knockdown of top predicted TFs affects healthy-failed repair gene expression. a. Heatmap of select differentially expressed genes by DESeq2 analysis of RNA-seq in NT and *CREB5* siRNA-treated RPTECs. **b.** Heatmap of select differentially expressed genes by DESeq2 analysis of RNA-seq in NT and *HIVEP2* siRNA-treated RPTECs. **c.** Heatmap of select differentially expressed genes by DESeq2 analysis of RNA-seq in NT and *PPARA* siRNA-treated RPTECs. Comparisons for **a-c** performed between n = 3 independent non-targeting control samples and n = 3 independent samples treated with siRNA targeting *CREB5*, *HIVEP2*, or *PPARA*, respectively.

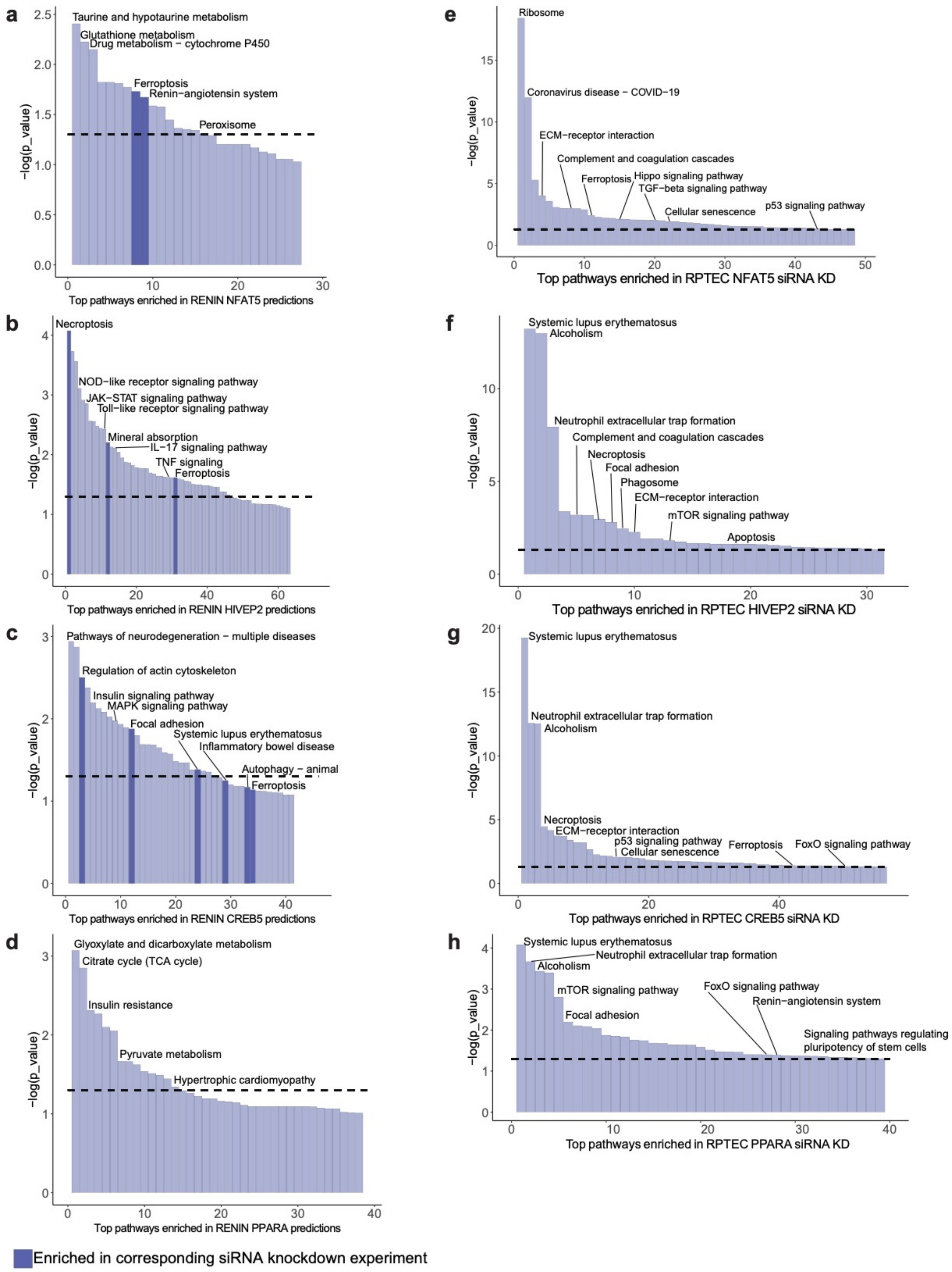
a
Taurine and hypotaurine metabolism
Glutathione metabolism
Drug metabolism − cytochrome P450
Ferroptosis
Renin−angiotensin system
Peroxisome
−log(p_value)
Top pathways enriched in RENIN NFAT5 predictions
b
Necroptosis
NOD−like receptor signaling pathway
JAK−STAT signaling pathway
Toll−like receptor signaling pathway
Mineral absorption
IL−17 signaling pathway
TNF signaling
Ferroptosis
−log(p_value)
Top pathways enriched in RENIN HIVEP2 predictions
c
Pathways of neurodegeneration − multiple diseases
Regulation of actin cytoskeleton
Insulin signaling pathway
MAPK signaling pathway
Focal adhesion
Systemic lupus erythematosus
Inflammatory bowel disease
Autophagy − animal
Ferroptosis
−log(p_value)
Top pathways enriched in RENIN CREB5 predictions
d
Glyoxylate and dicarboxylate metabolism
Citrate cycle (TCA cycle)
Insulin resistance
Pyruvate metabolism
Hypertrophic cardiomyopathy
−log(p_value)
Top pathways enriched in RENIN PPARA predictions
e
Ribosome
Coronavirus disease − COVID−19
ECM−receptor interaction
Complement and coagulation cascades
Ferroptosis
Hippo signaling pathway
TGF−beta signaling pathway
Cellular senescence
p53 signaling pathway
−log(p_value)
Top pathways enriched in RPTEC NFAT5 siRNA KD
f
Systemic lupus erythematosus
Alcoholism
Neutrophil extracellular trap formation
Complement and coagulation cascades
Necroptosis
Focal adhesion
Phagosome
ECM−receptor interaction
mTOR signaling pathway
Apoptosis
−log(p_value)
Top pathways enriched in RPTEC HIVEP2 siRNA KD
g
Systemic lupus erythematosus
Neutrophil extracellular trap formation
Alcoholism
Necroptosis
ECM−receptor interaction
p53 signaling pathway
Cellular senescence
Ferroptosis
FoxO signaling pathway
−log(p_value)
Top pathways enriched in RPTEC CREB5 siRNA KD
h
Systemic lupus erythematosus
Neutrophil extracellular trap formation
Alcoholism
mTOR signaling pathway
Focal adhesion
FoxO signaling pathway
Renin−angiotensin system
Signaling pathways regulating pluripotency of stem cells
−log(p_value)
Top pathways enriched in RPTEC PPARA siRNA KD
Enriched in corresponding siRNA knockdown experiment

Supplementary Figure 22. Pathway analysis of siRNA knockdown treatment. a-d. KEGG pathway enrichment in RENIN-predicted **(a)** NFAT5, **(b)** HIVEP2, **(c)** CREB5, and **(d)** PPARA target gene set. Dotted line corresponds to p value of 0.05. Dark purple bars correspond to pathways enriched in set of differentially expressed genes between non-targeting control and corresponding TF (*NFAT5*, *HIVEP2*, *CREB5*, and *PPARA*) siRNA-treated RPTECs. **e-h.** KEGG pathway enrichment in differentially expressed gene set between NT and **(e)** *NFAT5*, **(f)** *HIVEP2*, **(g)** *CREB5*, and **(h)** *PPARA* siRNA treatment.

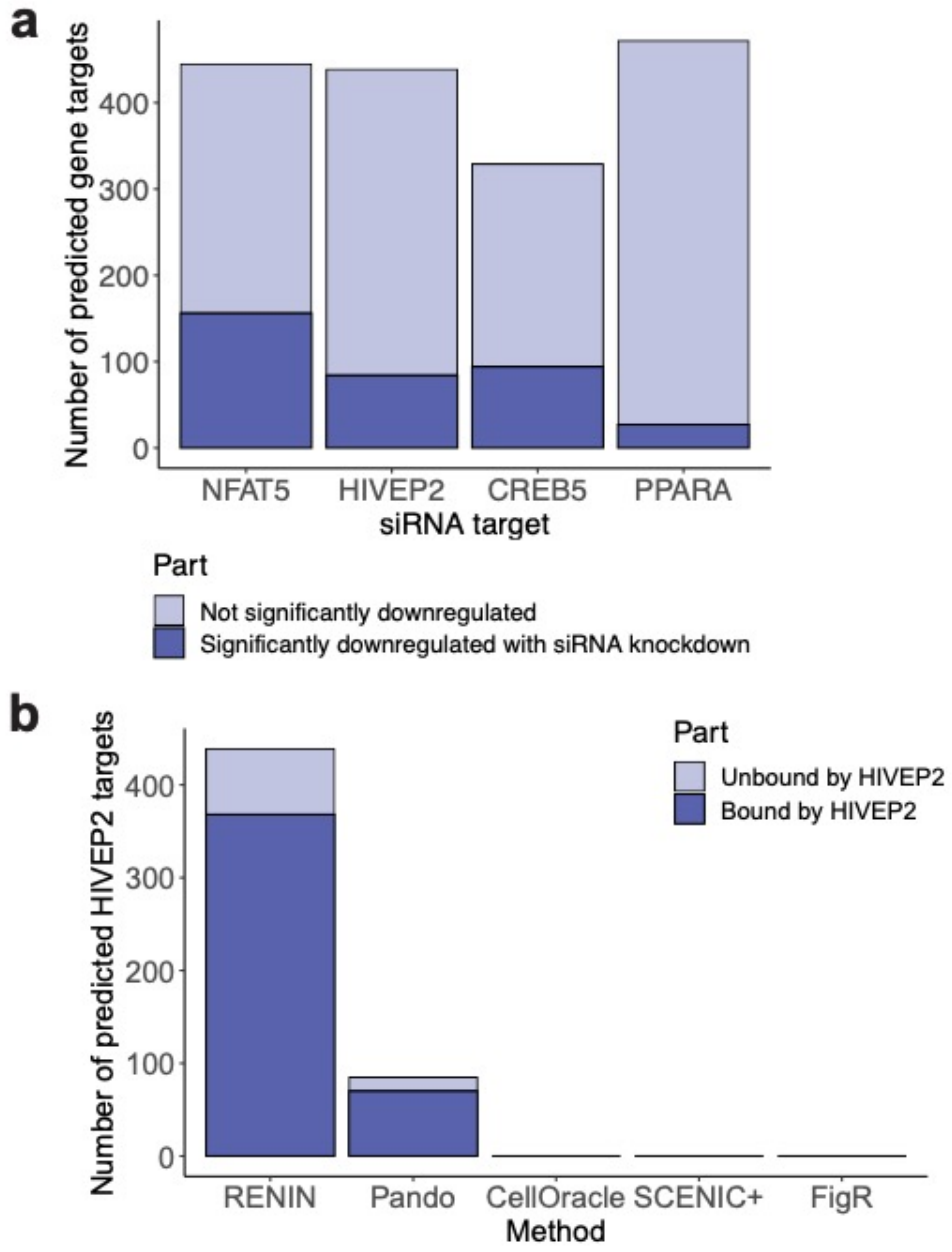

Supplementary Fig. 23. Proportions of predicted TF targets that are experimentally validated by siRNA knockdown and CUT&RUN. a. NFAT, HIVEP2, CREB5, and PPARA predicted targets (light purple) and the proportion that is significantly downregulated (adjusted p value < 0.05) with siRNA knockdown in RPTECs (dark purple). **b.** Number of predicted HIVEP2 targets by each method, separated into target genes that were bound versus unbound on HIVEP2 CUT&RUN-seq performed on n = 2 independent RPTEC samples. 368/439 RENIN-predicted targets and 70/85 Pando-predicted targets were bound by HIVEP2 assessed by CUT&RUN-seq on RPTEC culture. SCENIC+ does not predict any H-FR genes to be HIVEP2 targets. CellOracle and FigR do not use motif databases that include HIVEP2 motif necessary for modeling. Source data are provided in the Source Data file.

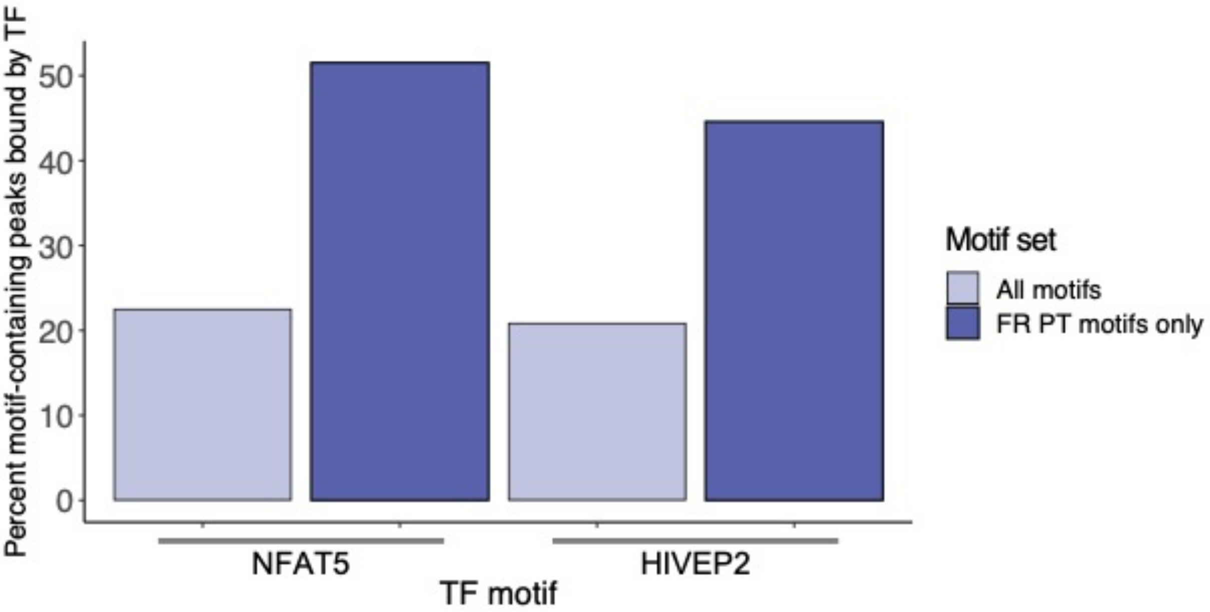

Supplementary Figure 24. NFAT5 and HIVEP2 motif-containing peaks bound in RPTEC CUT&RUN. a. 3,945 of 17,499 NFAT5 motif-containing peaks (22.5%) are bound by NFAT5. 795 of 1,543 NFAT5 motif-containing FR PT DARs (51.5%) are bound by NFAT5. **b.** 2,664 of 12,759 HIVEP2 motif-containing peaks (20.9%) are bound by HIVEP2. 697 of 1,563 HIVEP2 motif-containing FR PT DARs (44.6%) are bound by HIVEP2. Source data are provided in the Source Data file.

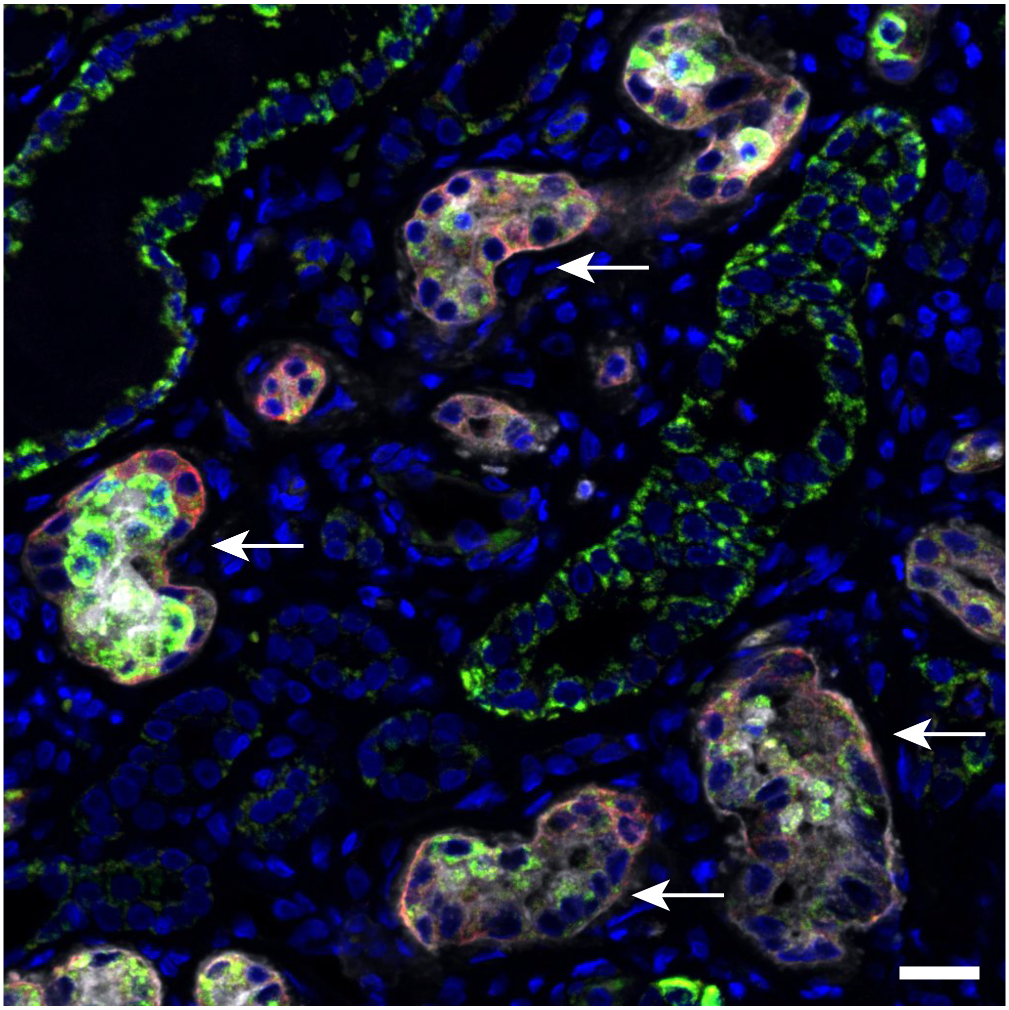

Supplementary Figure 25. VCAM1 and NFAT5 colocalization in ESRD kidney. Immunofluorescent labeling of NFAT5 (green) and VCAM1 (red) in adult human kidney. DAPI (blue) is a nucleus marker and LTL (white) is a proximal tubule marker. Arrows denote examples of NFAT+, VCAM1+ proximal tubules. Representative image of n = 2 independently analyzed ESRD samples. Sample clinical data in Source Data. Scale bar is 50 μm in length.

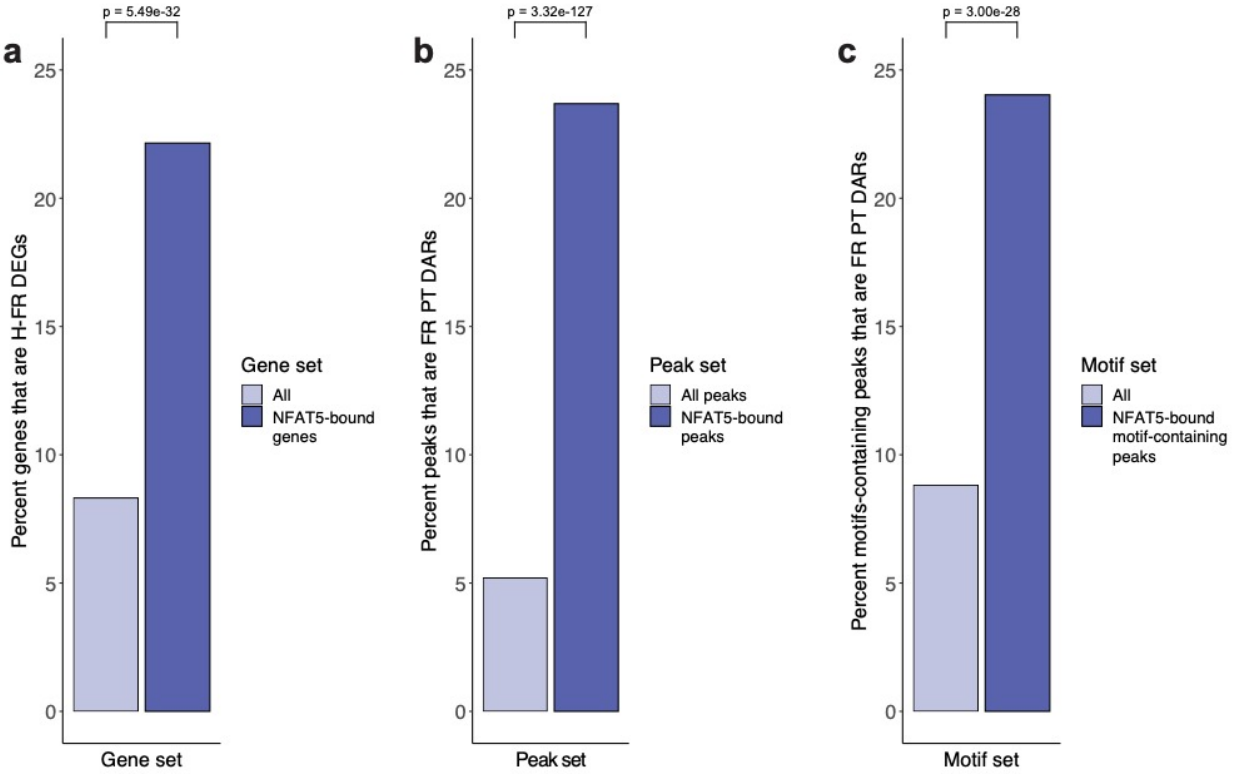

Supplementary Figure 26. H-FR genes and regulatory elements are overrepresented in NFAT5-bound peaks in whole kidney CUT&RUN. a. 1,519 of 18,262 genes (8.3%) are H-FR DEGs. 237 of 1,070 NFAT5-bound genes are H-FR DEGs. **b.** 10,082 of 193,787 (5.2%) peaks have increased accessibility in FR PT cells (FR PT DARs). 437 of 1,845 NFAT5-bound peaks are FR PT DARs (23.7%). **c.** 1,543 of 17,499 NFAT5 motif-containing peaks are FR PT DARs (8.8%). 198 of 824 NFAT5-bound, NFAT5 motif-containing peaks are FR PT DARs (24%). P values calculated by Fisher's exact test. Source data are provided in the Source Data file.
