# Supplementary material for: Predicting proximal tubule failed repair drivers through regularized regression analysis of single cell multiomic sequencing
Source: Nat Commun. 2024 Feb 12;15:1291. doi: 10.1038/s41467-024-45706-0 (PMC10861555; doi:10.1038/s41467-024-45706-0)
Supplement: Supplementary file 3 — Description of Additional Supplementary Files [file 41467_2024_45706_MOESM3_ESM.pdf]

Description of Additional Supplementary Files:

Supplementary Dataset 1: Cell type-specific differentially expressed genes identified by Seurat's FindMarkers function, with a named sheet for each cell type. Columns are the gene name, the unadjusted Wilcoxon rank sum p-value (p_val), log2 fold change of expression in the cell type versus other cells (avg_log2FC), proportion of cells in the cell type with expression of that row's gene (pct.1), the proportion of other cells in the dataset with expression of that row's gene (pct.2), and Bonferroni-adjusted p-value (p_val_adj).

Supplementary Dataset 2: Predicted cell type-specific cis-regulatory elements and their targets. Columns denote the peak identified as a cis-regulatory element (peak), the target gene (gene), predicted regulatory coefficients (regulatory coefficient), and cell type in which the relationship was predicted (celltype).

Supplementary Dataset 3: Genes differentially expressed between healthy proximal tubule (clusters PCT and PST) and failed repair proximal tubule (cluster KIM1+ PT). Columns are the gene name, the unadjusted Wilcoxon rank sum p-value (p_val), log2 fold change of expression in the cell type versus other cells (avg_log2FC), proportion of cells in the cell type with expression of that row's gene (pct.1), the proportion of other cells in the dataset with expression of that row's gene (pct.2), Bonferroni-adjusted p-value (p_val_adj), and whether the gene could be modeled based on our genome annotation (Modeled).

Supplementary Dataset 4: Overlap between CKD-associated differentially methylated regions (DMR) and all called peaks in our single cell multiome dataset (sheet: All peaks – DMR overlap) and RENIN-called cis-regulatory elements (sheet: RENIN CREs – DMR overlap). Each sheet contains the peaks that were found to overlap CKD-associated DMRs with findOverlaps.

Supplementary Dataset 5: Predicted regulatory scores of modeled transcription factors on genes differentially expressed between healthy (PCT and PST) and failed repair (KIM1+ PT) PT clusters. Each page corresponds to the number of cells per pseudocell targeted (1, 5, and 10). Columns are the transcription factor name (TF name) and cognate motif ID (Motif), along with the estimated score (Score) and standard error (SE). Scores are negative if the transcription factor is predicted to upregulate a failed repair expression profile or downregulate a healthy expression profile and positive otherwise.

Supplementary Dataset 6: Predicted regulatory scores of modeled transcription factors on genes differentially expressed between healthy (PCT and PST) and failed repair (KIM1+ PT) PT clusters with motif enrichment-based approach. Columns are the transcription factor name (TF name) and cognate motif ID (Motif), along with the estimated score (Score) and standard error (SE). Scores are

negative if the transcription factor is predicted to upregulate a failed repair expression profile or downregulate a healthy expression profile and positive otherwise.

Supplementary Dataset 7: Calculated AICs for each gene that could be modeled with all predicted cisregulatory elements versus with promoter peaks only. Columns are the gene name (Gene), the AIC for the TFs-gene model generated when modeling with all predicted CREs (All CREs), the AIC for the TFs-gene model generated when modeling with promoter peaks only (Prom only), and the difference in AIC (diff).

Supplementary Dataset 8: Predicted regulatory scores of modeled transcription factors on genes differentially expressed between healthy (PCT and PST) and failed repair (KIM1+ PT) PT clusters. Each page corresponds to the results of modeling gene regulatory networks in each of the 5 tested datasets. Columns are the transcription factor name (TF name) and cognate motif ID (Motif), along with the estimated score (Score) and standard error (SE). Scores are negative if the transcription factor is predicted to upregulate a failed repair expression profile or downregulate a healthy expression profile and positive otherwise.
