# Supplementary material for: Predicting proximal tubule failed repair drivers through regularized regression analysis of single cell multiomic sequencing
Source: Nat Commun. 2024 Feb 12;15:1291. doi: 10.1038/s41467-024-45706-0 (PMC10861555; doi:10.1038/s41467-024-45706-0)
Supplement: Supplementary file 12 — Reporting Summary [file 41467_2024_45706_MOESM12_ESM.pdf]

nature portfolio

Corresponding author(s): Benjamin D. Humphreys

Last updated by author(s): Dec 23, 2023

Reporting Summary

Nature Portfolio wishes to improve the reproducibility of the work that we publish. This form provides structure for consistency and transparency in reporting. For further information on Nature Portfolio policies, see our Editorial Policies and the Editorial Policy Checklist.

Statistics

For all statistical analyses, confirm that the following items are present in the figure legend, table legend, main text, or Methods section.

n/a	Confirmed	
☐	☒	The exact sample size (*n*) for each experimental group/condition, given as a discrete number and unit of measurement
☐	☒	A statement on whether measurements were taken from distinct samples or whether the same sample was measured repeatedly
☐	☒	The statistical test(s) used AND whether they are one- or two-sided<br>*Only common tests should be described solely by name; describe more complex techniques in the Methods section.*
☐	☒	A description of all covariates tested
☐	☒	A description of any assumptions or corrections, such as tests of normality and adjustment for multiple comparisons
☐	☒	A full description of the statistical parameters including central tendency (e.g. means) or other basic estimates (e.g. regression coefficient) AND variation (e.g. standard deviation) or associated estimates of uncertainty (e.g. confidence intervals)
☐	☒	For null hypothesis testing, the test statistic (e.g. *F*, *t*, *r*) with confidence intervals, effect sizes, degrees of freedom and *P* value noted<br>*Give P values as exact values whenever suitable.*
☒	☐	For Bayesian analysis, information on the choice of priors and Markov chain Monte Carlo settings
☐	☒	For hierarchical and complex designs, identification of the appropriate level for tests and full reporting of outcomes
☒	☐	Estimates of effect sizes (e.g. Cohen's *d*, Pearson's *r*), indicating how they were calculated

Our web collection on statistics for biologists contains articles on many of the points above.

Software and code

Policy information about availability of computer code

Data collection	Generation of feature-barcode matrices from FASTQ files was done with 10X Cell Ranger Arc 2.0 for the multiome data. Cell Ranger 6.0 and Cell Ranger ATAC 2.0 were used for the split RNA- and ATAC-seq datasets.
Data analysis	Data analysis used open source software packages: Seurat (4.0.2), Signac (1.2.1), MACS2 (2.2.7.1), CellBender (0.2.0), AMULET (1.1), ArchR (1.0.1), DoubletFinder (2.0.3), Harmony (0.1.0), chromVARmotifs (0.2.0), chromVAR (1.14.0), BSgenome.Mmusculus.UCSC.hg38 (1.4.3), BSgenome.Mmusculus.UCSC.mm10 (1.4.3), GenomicRanges (1.44.0) Cutadapt (2.8), Bowtie2 (2.3.5.1), samtools (1.9), DeepTools (3.5.0), STAR (2.7.9a), Subread:featureCount (2.0.3), DESeq2 (1.32.0), UCSC utilities (1.04.00), LDSC (1.0.1). For comparison with other methods, packages used: cicero (1.3.9), FigR (0.1.0), SCENIC+ (1.0.1), pycisTopic (1.0.3), scMega (0.2.0), MOJITOO (1.0), Pando (1.0.3), CellOracle (0.12.1). All custom analysis code written for this manuscript is publicly available on Zenodo at https://zenodo.org/record/7459341. The RENIN package to run the regression analysis is available on GitHub at https://www.github.com/nledru/RENIN.

For manuscripts utilizing custom algorithms or software that are central to the research but not yet described in published literature, software must be made available to editors and reviewers. We strongly encourage code deposition in a community repository (e.g. GitHub). See the Nature Portfolio guidelines for submitting code & software for further information.

Data

Policy information about availability of data

All manuscripts must include a data availability statement. This statement should provide the following information, where applicable:

- Accession codes, unique identifiers, or web links for publicly available datasets
- A description of any restrictions on data availability
- For clinical datasets or third party data, please ensure that the statement adheres to our policy

The datasets generated in this study have been deposited in GEO under accession codes GSE220289. A processed Seurat R object for the multiome dataset are available at Zenodo under record number 10444715. Source data are provided with this paper. Previously generated datasets that were analyzed during the current study are available in GEO under accession codes GSE151302 and GSE195443. The results here are also in part based upon data generated by KPMP: DK133081, DK133091, DK133092, DK133093, DK133095, DK1330971, DK114866, DK114908, DK133090, DK133113, DK133766, DK133768, DK114907, DK114920, DK114923, DK114933, DK114886. Data downloaded on 3/24/2023 from https://www.kpmp.org. The GRCh38-2020-A-2.0.0 reference genome, released on May 3, 2021, was downloaded from 10X Genomics. Source data are provided with this paper.

Research involving human participants, their data, or biological material

Policy information about studies with human participants or human data. See also policy information about sex, gender (identity/presentation), and sexual orientation and race, ethnicity and racism.

Reporting on sex and gender	Findings do not apply to one sex or gender, and neither sex nor gender were considered in study design. Gender data were not collected. We were interested in studying genetic regulation underlying chronic kidney disease, with the hypothesis that mechanisms we identified in our dataset with both male and female samples would be shared across sex. So sex- and gender-based analyses were not included in this manuscript.
Reporting on race, ethnicity, or other socially relevant groupings	We did not use socially constructed or socially relevant categorizations in this manuscript on the basis of race, ethnicity, or other social grouping for any analysis in this manuscript. For the model training and other analysis, all samples were analyzed jointly.
Population characteristics	Clinical meta data listed in Source Data for experiments using samples from patients, including those from which sequencing data were generated.
Recruitment	Nephrectomy- and biopsy-derived samples were collected from 7 individuals for multiome sequencing. Biopsy-derived samples were collected from 3 individuals for split single nucleus RNA-seq and ATAC-seq. Samples were used with informed consent. For recruitment, all eligible patients were screened, but all samples are subject to any bias connected with being willing to donate tissue and/or from families willing to let their relation's organs be used for transplant. There was no participant compensation.
Ethics oversight	All participants provided written informed consent in accordance with the Declaration of Helsinki. All appropriate consents, including to publish, have been obtained in the original consent document. This research complies with all relevant ethical regulations and has been approved by the Washington University Institutional Review Board. Samples were collected under established IRB protocols approved by the Mass General Brigham Human Research Committee, Washington University Institutional Review Board, and Wake Forest University Institutional Review Board.

Note that full information on the approval of the study protocol must also be provided in the manuscript.

Field-specific reporting

Please select the one below that is the best fit for your research. If you are not sure, read the appropriate sections before making your selection.

[x] Life sciences [ ] Behavioural & social sciences [ ] Ecological, evolutionary & environmental sciences

For a reference copy of the document with all sections, see nature.com/documents/nr-reporting-summary-flat.pdf

Life sciences study design

All studies must disclose on these points even when the disclosure is negative.

Sample size	No sample size calculation was performed. Samples from 7 individuals were processed to maximize nucleus recovery and sequencing. Nuclei recovered averaged roughly 3000-7000 nuclei per 10X Chromium Controller lane, and additional lanes were prepared given sufficient starting tissue amount. The number of nuclei analyzed in this study was sufficient to draw the conclusions described in the manuscript.
Data exclusions	ArchR 1.0.1 was used for preliminary filtering (TSSEnrichment >= 4, BlacklistRatio <= 0.01, NucleosomeRatio <= 4, nFrags >= 3000, and nFrags <= 100000). Seurat 4.0.2 was also used for preliminary filtering (nCount_ATAC > 3000, nCount_ATAC < 100000, nFeature_ATAC > 1000, nCount_RNA < 50000, nCount_RNA > 1000, nFeature_RNA > 500, percent.mt< 5, percent.rps< 2, percent.rpl < 2), and cell barcodes passing both sets of filtering criteria were retained. Three small clusters with few barcodes, poor sequencing depth, and composed primarily of one sample were removed. Predicted doublets by either AMULET or DoubletFinder were also removed.

Replication	For single cell sequencing data, samples were processed individually. Gene expression and chromatin accessibility profiles were similar across samples. Single cell modeling experiments were repeated in n=3-5 trials with different RNG seeds with successful replication of TF ranks and similar scores. Simulations were repeated in n=3 independent trials with successful replication of simulated trajectory direction. For the cell type analyses with our single cell multiomic dataset, all studied cell types were present in all samples. Cell type analyses for marker gene expression and TF ranks and scores were similar over n=3 trials. CUT&RUN for histone modifications, siRNA knockdown, and RNA-seq were performed on n=3 RPTEC cell culture samples. CUT&RUN for transcription factors were performed on n=2 RPTEC cell culture or human whole kidney samples for peak calling.
Randomization	Randomization is not relevant to this study because there was a single experimental group with samples from 7 individuals.
Blinding	No blinding was used during data collection or analysis. It was not considered necessary for the study, because measurements could not be affected by the operator.

Reporting for specific materials, systems and methods

We require information from authors about some types of materials, experimental systems and methods used in many studies. Here, indicate whether each material, system or method listed is relevant to your study. If you are not sure if a list item applies to your research, read the appropriate section before selecting a response.

Materials & experimental systems

n/a	Involved in the study	
☐	☒	Antibodies
☐	☒	Eukaryotic cell lines
☒	☐	Palaeontology and archaeology
☒	☐	Animals and other organisms
☒	☐	Clinical data
☒	☐	Dual use research of concern
☒	☐	Plants

Methods

n/a	Involved in the study	
☒	☐	ChIP-seq
☒	☐	Flow cytometry
☒	☐	MRI-based neuroimaging

Antibodies

Antibodies used	The following primary antibodies were used for CUT&RUN: H3K27ac antibody (Epicypher, 13-0045), H3K4me3 antibody (Epicypher, 13-0041), NFAT5 antibody (Thermo Fisher Scientific, PA1-023), HIVEP2 antibody (Thermo Fisher Scientific, PA5-100756), rabbit IgG negative control antibody (Epicypher, 13-0042). The following antibodies were used for immunofluorescence studies: primary - NFAT5 (Thermo Fisher Scientific, PA1-023) and VCAM1 (Abcam, ab134047) and secondary - Alexa Fluor 488-conjugated Donkey anti-rabbit (Thermo Fisher Scientific, A-21206), anti-rabbit ImmPRESS secondary (Vector Laboratories, MP-7401-15), Tyramide-AF488 (Thermo Fisher Scientific, B40953). Biotinylated Lotus Tetragonolobus Lectin (LTL) (Vector Laboratories, B-1325) and Alexa Fluor 647-conjugated strepavidin (Thermo Fisher Scientific, S32357) were also used.

Validation	Primaries<br>Antigen; species; application; link; validation statement from company's website<br>H3K27ac; mouse; ChIP, ChIP-Seq, L, IF; https://www.epicypher.com/products/antibodies/snap-chip-certified-antibodies/histone-h3k27ac-antibody-snap-chip-certified; "This antibody meets EpiCypher's "SNAP-ChIP® Certified" criteria for specificity and efficient target enrichment in a ChIP experiment (<20% cross-reactivity across the panel, >5% recovery of target input)."<br>H3K4me3; rabbit; CUT&RUN, ChIP, ICC/IF, WB; https://www.epicypher.com/products/antibodies/snap-chip-certified-antibodies/histone-h3k4me3-antibody-snap-chip-certified-cutana-cut-run-compatible; This H3K4me3 (histone H3 lysine 4 trimethyl) antibody meets EpiCypher's lot-specific SNAP-Certified™ criteria for specificity and efficient target enrichment in CUT&RUN. This requires <20% cross-reactivity to related histone PTMs determined using the SNAP-CUTANA™ K-MetStat Panel of spike-in controls<br>Negative control IgG; rabbit; negative control for CUT&RUN, CUT&Tag; https://www.epicypher.com/products/nucleosomes/snap-cutana-spike-in-controls/cutana-rabbit-igg-cut-run-negative-control-antibody; CUT&RUN was performed as described in Figure 3. Heatmaps show H3K4me3 peaks relative to IgG antibody in aligned rows ranked by intensity (top to bottom) and colored such that red indicates high localized enrichment and blue denotes background signal. H3K4me3 antibody showed expected enrichment around the TSS, while the IgG antibody displayed minimal background, as expected.<br>NFAT5; rabbit; IHC, ICC/IF, IP, ChIP, WB; https://www.thermofisher.com/antibody/product/NFAT5-Antibody-Polyclonal/PA1-023; This Antibody was verified by Knockdown to ensure that the antibody binds to the antigen stated.<br>VCAM1; rabbit; WB, IP, IHC-P, Flow Cyt (Intra), ICC/IF, Indirect ELISA; rabbit; https://www.abcam.com/products/primary-antibodies/vcam1-antibody-epr5047-ab134047.html; ab134047 was shown to react with VCAM1 in treated wild-type A549 cells in Western blot with loss of signal observed in treated VCAM1 knockout cell line ab273758<br>HIVEP2; rabbit; WB, Flow Cytometry; https://www.thermofisher.com/antibody/product/HIVEP2-Antibody-Polyclonal/PA5-100756;<br>anti-rabbit secondary; donkey; https://www.thermofisher.com/antibody/product/Donkey-anti-Rabbit-IgG-H-L-Highly-Cross-Adsorbed-Secondary-Antibody-Polyclonal/A-21206; Western blot analysis of HIVEP2 in extracts from various samples. Samples were incubated in HIVEP2 polyclonal antibody. Western blot analysis of HIVEP2 in immunizing recombinant protein (left lane: treated with the antigen-specific peptide). Samples were incubated with HIVEP2 polyclonal antibody.<br>anti-rabbit ImmPRESS secondary; horse; https://vectorlabs.com/products/enzyme-polymer/immpress-hrp-horse-anti-rabbit-igg<br>Alexa Fluor 488-conjugated Tyramide; https://www.thermofisher.com/order/catalog/product/B40953<br>Biotinylated LTL; https://vectorlabs.com/products/glycobiology/biotinylated-lotus-tetragonolobus-lectin-ltl<br>Alexa Fluor 647-conjugated streptavidin; https://www.thermofisher.com/order/catalog/product/S32357

Eukaryotic cell lines

Policy information about cell lines and Sex and Gender in Research

Cell line source(s)	Human primary proximal tubular cells were obtained from Lonza (CC-2553).
Authentication	Not authenticated by authors.
Mycoplasma contamination	Cells were negative for Mycoplasma contamination.
Commonly misidentified lines<br>(See ICLAC register)	No commonly misidentified cell lines were used.

Plants

Seed stocks	*Report on the source of all seed stocks or other plant material used. If applicable, state the seed stock centre and catalogue number. If plant specimens were collected from the field, describe the collection location, date and sampling procedures.*
Novel plant genotypes	*Describe the methods by which all novel plant genotypes were produced. This includes those generated by transgenic approaches, gene editing, chemical/radiation-based mutagenesis and hybridization. For transgenic lines, describe the transformation method, the number of independent lines analyzed and the generation upon which experiments were performed. For gene-edited lines, describe the editor used, the endogenous sequence targeted for editing, the targeting guide RNA sequence (if applicable) and how the editor was applied.*
Authentication	*Describe any authentication procedures for each seed stock used or novel genotype generated. Describe any experiments used to assess the effect of a mutation and, where applicable, how potential secondary effects (e.g. second site T-DNA insertions, mosiacism, off-target gene editing) were examined.*
